# Determinants of outpatient service use among Orang Asli in Malaysia using Andersen’s Behavioural Model

**DOI:** 10.1371/journal.pone.0340502

**Published:** 2026-01-22

**Authors:** Iqbal Ab Rahim, Nur Elina Abdul Mutalib, Sivaraj Raman, Devi Shantini Rata Mohan, Awatef Amer Nordin, Suhana Jawahir, Sarah Nurain Mohd Noh, Jabrullah Ab Hamid, Hamizatul Akmal Abd Hamid, Tania Gayle Robert Lourdes, Thamil Arasu Saminathan, Mohd Hatta Abdul Mutalip, Adilius Manual

**Affiliations:** 1 Centre for Health Equity Research, Institute for Health Systems Research, National Institutes of Health, Ministry of Health Malaysia, Shah Alam, Selangor, Malaysia; 2 Centre for Health Economics Research, Institute for Health Systems Research, National Institutes of Health, Ministry of Health Malaysia, Shah Alam, Selangor, Malaysia; 3 Centre for Non-communicable Disease Research, Institute for Public Health, National Institutes of Health, Ministry of Health Malaysia, Shah Alam, Selangor, Malaysia; 4 Centre for Communicable Disease Research, Institute for Public Health, National Institutes of Health, Ministry of Health Malaysia, Shah Alam, Selangor, Malaysia; 5 Faculty of Medicine and Health Sciences, Universiti Malaysia Sabah, Jalan UMS, Kota Kinabalu, Sabah, Malaysia; Xiamen University - Malaysia Campus: Xiamen University - Malaysia, MALAYSIA

## Abstract

**Background:**

Indigenous populations, including the Orang Asli in Malaysia, experience persistent health disparities due to historical, socioeconomic, geographic, and cultural barriers. Despite government initiatives to improve access, significant gaps remain, and limited nationwide data hinder policy development. This study examines the prevalence and determinants of outpatient healthcare utilisation among the Orang Asli.

**Methods:**

This study utilised data from the Orang Asli Health Survey, a nationwide cross-sectional survey of Orang Asli communities in Peninsular Malaysia, with 89.8% response rate. Andersen’s Behavioural Model was applied in the analysis to assess the predisposing, enabling, and health need factors influencing outpatient healthcare use among the adult (aged 18 and over) population. Weighted descriptive statistics and logistic regression were used to examine outpatient healthcare utilisation and its determinants. Analyses were performed in STATA 18.

**Results:**

The overall prevalence of outpatient service utilisation in the past 12 months was 17.9%. Higher utilisation was observed among females, urban residents, and the Senoi and Negrito tribes. Determinants of outpatient use included female (adjusted odds ratio [aOR]: 1.64, 95% CI: 1.31–2.06), urban locality (aOR: 2.39, 95% CI: 1.15–4.96), Senoi (aOR 2.66; 95% CI: 1.52–4.64) and Negrito (aOR: 3.91, 95% CI: 2.01–7.60) tribes, unemployment (aOR: 1.27, 95% CI: 1.11–1.46), recent acute health problems (aOR: 2.17, 95% CI: 1.68–2.81), fair to very poor self-rated health (aOR: 2.30, 95% CI: 1.39–3.79), and presence of one (aOR: 2.90, 95% CI: 2.00–4.21) or two or more non-communicable diseases (NCD) (aOR: 4.63, 95% CI: 2.89–7.41). Interaction effects indicated lower outpatient use among Senoi and Negrito adults with poor self-rated health compared to other groups.

**Conclusion:**

Outpatient healthcare utilisation among Orang Asli adults was driven by gender, tribe, health needs, and NCDs. Improved access requires needs-based sensitive interventions and existing services optimisation. Follow-up studies are warranted to explore the underlying cultural behavioural aspects.

## Introduction

Indigenous populations, which constitute approximately 5% of the global population, consistently experience poorer health outcomes due to geographic location, historical marginalisation, socioeconomic disadvantages, and limited access to basic amenities and healthcare [1]. In Malaysia, the indigenous community consists of the Orang Asli in Peninsular Malaysia and the natives in Sabah and Sarawak. The Orang Asli, meaning “original people” in Malay, comprise about 0.8% of the Peninsular Malaysia population [2]. They consist of a diverse group of communities that are classified into three main ethnic groups: the Negrito, Senoi, and Proto-Malay, each with distinct languages, customs, and settlement patterns. The Negrito, the smallest group, generally inhabit the deep forested regions of northern Peninsular Malaysia; the Senoi, accounting for about 55% of the total Orang Asli, occupy the central highlands of Perak, Kelantan and Pahang; while the Proto-Malay typically reside in the coastal and lowland areas, with permanent settlements in Pahang, Johor, Negeri Sembilan and, Selangor [3].

Similar to indigenous populations elsewhere, the Orang Asli also face significant political, economic, and social challenges. These challenges not only influence their health outcomes but also raise broader concerns about the social justice, equity, and inclusivity in healthcare services. To mitigate these barriers, the Malaysian government has implemented various policies aimed at improving their socioeconomic and health status. In the 1970s, initial efforts focused on security and integration, which involved relocation and regroupment schemes known as *Rancangan Pengumpulan Semula* (RPS) [4,5]. The scheme aimed to consolidate scattered communities closer to state services and to facilitate agricultural development. Agencies such as the Department of Orang Asli Development (JAKOA) were subsequently established to support improvements in healthcare access, infrastructure improvements, and education services [6,7]. Despite these initiatives, substantial health disparities persist, with the Orang Asli experiencing higher rates of infant mortality, infectious diseases, and limited access to healthcare compared with the general population [8].

Geographic isolation remains a major barrier for the Orang Asli, as many settlements are in remote areas with limited transportation infrastructure, reducing timely access to healthcare facilities [5,9]. High travel costs and limited availability of healthcare facilities further discourage utilisation, particularly for routine or preventive services. Additionally, poverty and financial framework limit their ability to afford healthcare [10]. Cultural and linguistic factors also impede their access to healthcare. Many Orang Asli communities continue to rely on traditional healing practices and express mistrust towards modern medical services, shaped by past experiences of discrimination or inadequate, culturally insensitive care [11]. Low health literacy and language barriers can also hinder awareness of preventive healthcare and delays in treatment seeking, exacerbating existing health disparities [12].

Recognising these challenges, the Malaysian government has implemented policies since 1979 to improve healthcare accessibility [13]. For example, the Ministry of Health exempts indigenous individuals from admission charges and outpatient clinic registration fees in the public health facilities. While other health services, such as screenings, are not routinely exempted, waivers can be granted under Paragraph 16 (13 ) of the Fees (Medical) Order 1982 to reduce their financial burden [14]. Additionally, structured resettlement programs have been implemented to provide basic amenities, housing, access to electricity and water, schools and health services within the settlements, at the peripheries or in close proximity to township areas, thereby improving access [5,9]. While these efforts are commendable, resettlement initiatives continue to prompt debates, particularly regarding cultural preservation and whether such policies translate into improved healthcare utilisation and outcomes.

Currently, there is no nationwide data on the outpatient utilisation among the Orang Asli communities. Although the National Health and Morbidity Survey (NHMS) routinely included Orang Asli as part of its sampled population, the findings are unable to elicit detailed health status information specific to Orang Asli. Past studies are also mostly small-scaled and focused on specific sub-tribes and the health services provided [9,15–19]. These evidence gaps hinder the development of inclusive national health policies, especially those aimed at strengthening and tailoring primary health care interventions for this marginalised group [9,18,19]. Access to primary care services, usually represented by the use of outpatient services, is the public’s main point of contact with the healthcare system. It plays a key role in equity, cost containment, and care. Understanding patterns and determinants of outpatient healthcare utilisation can elucidate potential disparities, evaluate policy effectiveness, and interpret service use within a specific social and cultural context [20–22]. To address this gap, the Andersen’s Behavioural Model for Health Use can be applied to explain the outpatient healthcare utilisation among Orang Asli. This model was adopted to contextualise healthcare use as a sequential and conditional process influenced by three key determinants: predisposing factors (demographic and social), enabling factors (economic), and health need factors (health status) [23]. This study, therefore, aims to determine the prevalence, user characteristics, and determinants of outpatient use among Orang Asli adults in Malaysia.

## Materials and methods

### Study population, setting and participants

The Orang Asli Health Survey (OAHS) was conducted as a nationwide cross-sectional nationwide survey. OAHS applied a two-stage stratified sampling design, first by primary stratum (urban, fringe or rural locality), and subsequently by the three main tribes (Senoi, Proto-Malay, and Negrito). The sampling frame, obtained from JAKOA, was based on population data provided by JAKOA; it comprised 853 Orang Asli villages across Peninsular Malaysia, including Selangor, Perak, Kedah, Melaka, Negeri Sembilan, Johor, Kelantan, Terengganu, and Pahang.

Data collection was performed from June to September 2022 via face-to-face interviews using a structured, pre-tested questionnaire in the Malay language. To ensure consistency across sampling sites, the questionnaire was supplemented with manuals, a codebook, and a dictionary of common Orang Asli terminologies. At least one Orang Asli research assistant was included in each field data collection team to facilitate communication. Representatives from JAKOA were also present during the pre-data collection meetings with each village leader and during data collection to facilitate the participant recruitment process.

Eligible participants were individuals residing in non-institutionalised living quarters for at least two weeks prior to data collection. Those living in institutional settings such as old folks' homes, hostels, hotels and hospitals were excluded. Prior to interviewing, the survey’s objectives and the Patient Information Sheet (PIS) were explained. Written informed consent was obtained from all respondents or their guardians, with additional assent collected from participants aged seven to 18 years. For individuals with low literacy levels, the PIS was read aloud, and thumbprints were obtained in place of signatures.

Across 68 randomly selected villages, 4,378 living quarters (LQs) and 16,811 participants were identified as eligible. A non-replacement protocol was used for respondents who refused or were unavailable, with data collectors required to make at least three contact attempts before recording a non-response. Non-response was subsequently accounted for using a specific non-response adjustment factor incorporated into the final sample weighting formula to maintain representativeness without introducing replacement bias.

Overall, 4,141 LQs and 15,950 participants completed the survey, yielding a response rate of 89.8%. Further details on the methodology and sampling design have been published elsewhere [24].

### Target population for analysis

For the present analysis, only adults aged 18 years and over (n = 9,225) who fulfilled the inclusion criteria, provided informed consent, and completed the OAHS questionnaire were included. Analysis was conducted for all cases in the selected variables influencing the outpatient healthcare utilisation in the last 12 months, with covariates from different modules in the OAHS [24]. Since each module was designed for different age groups, restricting the sample to adults ensured greater consistency across variables and minimised missing data in subsequent regression.

### Variables

**Fig 1:**
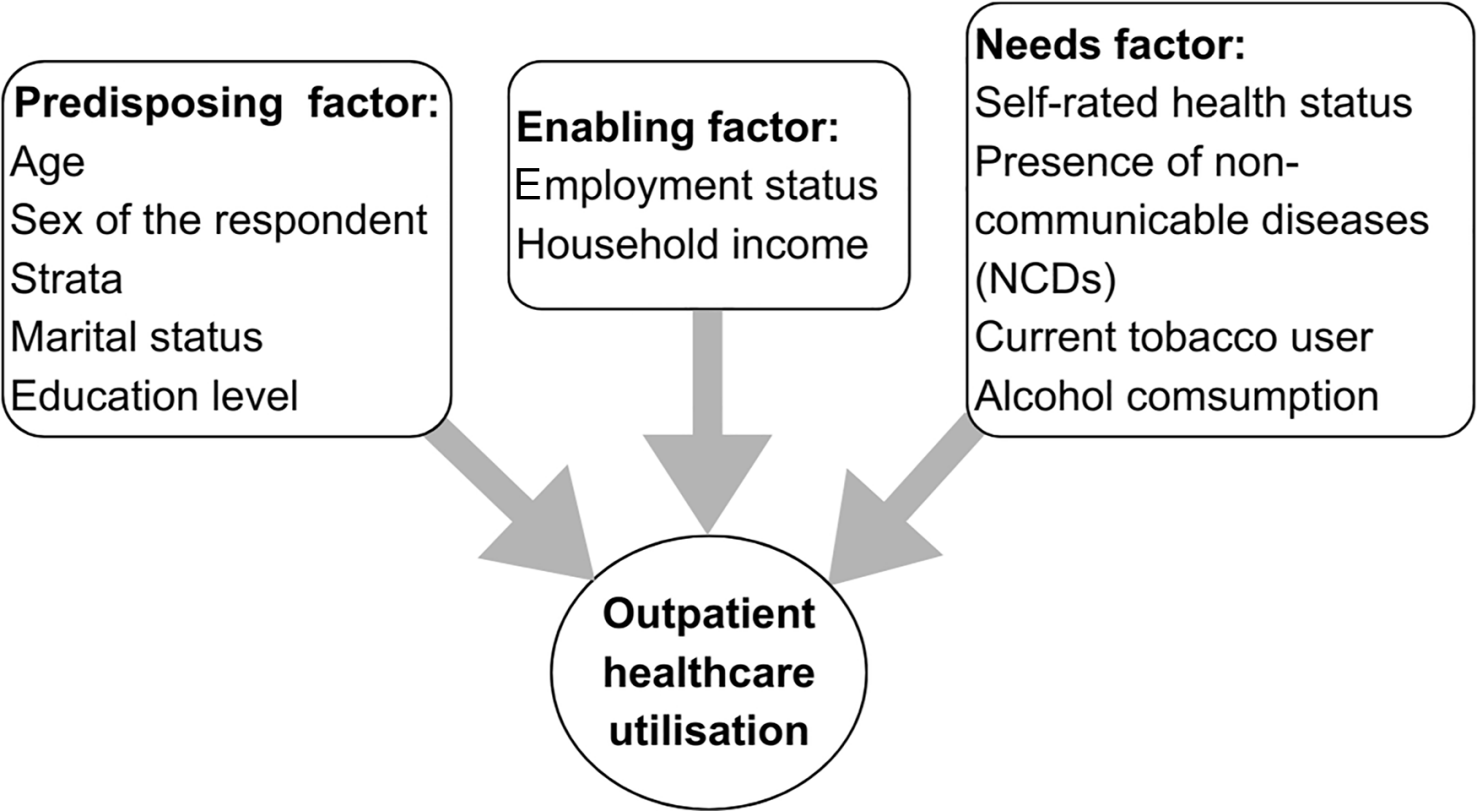
Variables grouped based on Andersen’s Behavioural Model in this study.

Fig 1 shows the independent variables grouped based on the factors (predisposing, enabling, and needs) to predict the dependent variable, health use, i.e., Orang Asli adults’ outpatient healthcare utilisation based on the conceptual framework Andersen’s Behavioural Model for Health Service Use [25].

#### Outcome variable.

Outpatient healthcare utilisation was measured by the question “In the last 12 months, did you get any outpatient care at clinics or hospitals?” with “yes” or “no” as the responses of choice. Those who answered “do not know” or “refuse to answer” were categorised as missing. Of the 9,225 adult respondents, about 1,878 responded that they have visited clinics or hospitals for outpatient care in the last 12 months. For outpatient utilisation, the recall period differs from the national health survey (2 weeks). The adjustment was made based on the insights found during the pre-testing of the questionnaire prior to the survey, as well as other studies involving indigenous communities in Australia and Canada [26,27]. Furthermore, participants were also asked about the type of services or treatments obtained for the outpatient services, for example, common illnesses, antenatal, vaccination, medical refills, health screening and others, to further verify the service use in the last 12 months.

#### Independent variables.

The predisposing factors were sex, strata, Orang Asli tribes, age, marital status, and education level. Strata were categorised as “urban”, “fringe” or “remote” based on JAKOA’s classification [3,6]. Orang Asli tribes analysed in this study were the three major tribes categorised as: Senoi, Proto-Malays, and Negrito. Age, initially a continuous variable measured in years, was categorised into three groups: “18-39”, “40-59”, and “60 and over” based on the age distribution pattern. Marital status of the respondents was categorised as either “Single or widowed or divorced” or “Married or Living with a partner” for this study. Education level was categorised as “no formal education or never went to school” for those with no formal education or never attained any education or have finished or attended primary or secondary education as having “primary (finished/attended)” or “secondary (finished/attended)” education.

The enabling factors included in this study were employment status and household income. Employment was measured whether the individual was employed within one month prior to the interview, with “yes” or “no” as response choices and was categorised as “employed” or “unemployed”. Household income was anchored to the total monthly household income of the head of household, further categorised into households with total income “Malaysian Ringgit (MYR) 0–999” or “MYR 1,000 and over”.

Perceived and evaluated needs were included as measures for the need factors. Perceived needs included in this study were self-rated health (SRH) status and reported health problems. SRH status was assessed using a five-point scale (excellent, good, fair, poor, very poor) and the question, “How would you rate your health status?” In the analysis, the responses were grouped into two categories: “good to excellent” and “fair, poor to very poor”. Reported recent acute health problems were measured by the question, “In the last two weeks, did you experience any of the following health problems, such as fever, sore throat, difficulty in swallowing, running nose or blocked nose, cough, and other symptoms of acute health problems?”, with “yes” or “no” as response choices.

As for evaluated needs, it was assessed based on the presence of any of these non-communicable diseases (NCDs) that were self-reported or previously diagnosed by a healthcare practitioner: diabetes, hypertension, or hypercholesterolemia. Responses for these health conditions were further grouped as either “No NCD”, “1 NCD” or “2 or more NCDs”. Modifiable health risk behaviours were also included in the analysis, i.e., smoking status and alcohol consumption, with “yes” or “no” responses. For any usage of tobacco, an individual who currently uses any tobacco product during the interview period was considered a current tobacco user. For alcohol consumption habits, an individual who had consumed any alcoholic beverages in the last 12 months preceding the survey was considered a current alcohol drinker.

### Statistical analysis

Data analysis was conducted using STATA version 18 (Stata Corp, College Station, TX, USA) to analyse the descriptive statistics and regression analysis. Sampling weights were applied to account for the complex survey design and clustering. To account for the complex survey design (stratification and clustering), associations between categorical variables were tested using the Rao-Scott chi-square test, which produces an F-statistic with adjusted degrees of freedom. Unweighted counts, weighted population estimates and prevalence [and confidence intervals of 95% (95%CI)] are presented, and p-values <0.05 were considered statistically significant. Descriptive statistics were used to illustrate the Orang Asli adult respondents, as well as outpatient healthcare service users, for all the responses, including the missing values. Estimates with relative standard errors (RSE) greater than 30% were considered unreliable and were interpreted with caution [28]. Cases with missing values (n = 157, 1.7%), however, were excluded from logistic regression.

Univariate and multivariable logistic regression were employed to determine the associations between the predisposing, enabling, needs and health behaviours factors with the utilisation of outpatient services. For the final multivariable regression model, we applied a two-step approach for variable selection guided by both theoretical considerations (previous literature) and statistical criteria [29]. First, variables with a statistical significance *p*-value≤0.25 in the univariate regression analysis were included in the final multivariable regression analysis [30,31]. Variables for which univariate analysis had shown a statistically significant association with the outpatient healthcare utilisation were included in the preliminary model as covariates by stepwise selection. Other variables, which were considered relevant, age group and education level, from other literature, were included in the final multivariable model [32]. Secondly, the change-in-estimate approach (chest) was employed to identify potential confounders to be included in the final model [33,34]. In addition to age group and education level, the final model adjusted for sex, strata, tribe, marital status, employment status, recent acute health problems, self-rated health, presence of NCDs, alcohol use, and smoking status. The crude and adjusted odds ratios (ORs) and their respective confidence intervals (CIs) were calculated. *p*-values less than 0.05 were considered to denote statistically significant association in our study. Two-way interaction terms (especially for tribe-specific interactions) were tested in the regression model to examine the difference in the associations between the independent variables and the outcome variable. The goodness of fit was assessed using Archer-Lemeshow (where *p* > 0.05 indicates a good fit) and the area under the curve (AUC) value for receiver operating characteristic curves (where AUC ≥ 0.9 is excellent, 0.8–0.9 is good, 0.7–0.8 is fair and below 0.7 suggests limited predictive ability) to allow for proper interpretation of the regression model [31,35]. The multicollinearity was examined using the variance inflation factor (VIF), whereby VIF values greater than 10 indicate the presence of multicollinearity [36].

### Ethical approval and consent to participate

The OAHS study was registered under the National Medical Research Register (NMRR-19-3108-50999). Ethical approval for the study was granted by the Medical Research and Ethics Committee (MREC) of the Ministry of Health Malaysia (KKM/NIHSEC/P19-2592 (11) on December 18^th^, 2019). Funding for this survey was received from the Ministry of Health Research Grant, (NMRR-19-3108-50999). Approval from the Department of Orang Asli Development (JAKOA) Malaysia and relevant local authorities was obtained before the data collection was conducted. The tenets of the Declaration of Helsinki were followed during the study. Informed consent was obtained from respondents before the interviews. The authors declare no conflict of interest in any form. There is no conflict of interest with the funder, no influence in the design, data collection, data analysis, or the writing of the manuscript. All eligible participants were informed about the survey, and informed written consent was obtained from all potential respondents prior to conducting the survey interview and related assessments.

The de-identified data from the OAHS’s data custodian were accessed for research purposes on 1^st^ February 2024. The authors did not have access to any information that could identify individual participants at any point during or after data collection.

## Results

### Sociodemographic characteristics

Overall, a total of 9,225 respondents were included in this study, of the total 15,950 respondents, representing 108,295 Orang Asli adults in Malaysia. The adult respondents consist of 52.3% (95%CI: 39.10–65.24), 45.6% (95%CI: 32.71–59.18) and 2.0% (95%CI: 1.45–2.84) of Senoi, Proto-Malay and Negrito tribes, respectively. Most of the respondents resided in fringe [75.9% (95%CI:67.33–82.81)] and remote areas [23.0% (95%CI:1.30–31.41)]. Most adult respondents in this study belonged to the 18–39 years old age group [59.3% (95%CI: 56.27–62.17)]. More than 60% (95%CI:56.81–73.32) of the respondents have a household income less than MYR 999. About 40.8% (95%CI: 37.20–44.58) were unemployed during the data collection. Most of the Orang Asli adults did not smoke any tobacco products [67.1% (95%CI: 61.89–71.94)] or consumed alcoholic beverages [89.6% (95%CI: 85.30–92.69)] prior to the survey. The respondents’ characteristics are shown in Table 1.

**Table 1:** Characteristics of the Orang Asli respondents aged 18 years old and over (n = 9,225).

Characteristics	Unweighted counts	Estimated population	% (95% CI)
***Predisposing Factors***			
**Sex**			
Male	4,045	46,757	43.2 (41.23–45.15)
Female	5,180	61,538	56.8 (54.85–58.77)
**Strata**			
Urban	808	1,191	1.1 (0.62–1.95)
Fringe	4,323	82,203	75.9 (67.33–82.81)
Remote	4,094	24,901	23.0 (16.30–31.41)
**Tribe**			
Senoi	3,848	56,670	52.3 (39.10–65.24)
Proto Malay	3,259	49,422	45.6 (32.71–59.18)
Negrito	2,118	2,203	2.0 (1.45–2.84)
**Age group (years)**			
18–39	5,726	64,168	59.3 (56.27–62.17)
40–59	2,656	32,806	30.3 (28.14–32.53)
60 and over	843	11,322	10.5 (8.88–12.27)
**Marital status**			
Single/Divorced/Widowed	2,441	30,596	28.3 (24.66–32.15)
Married	6,762	77,226	71.3 (67.42–74.91)
Missing	22	472	0.4 (0.28–0.69)
**Education**			
No formal education/ Never went to school	2,331	28,291	26.1 (19.36–34.25)
Primary education (finished/attended)	3,884	44,131	40.8 (34.68–47.12)
Secondary education (finished/attended)	2,960	35,123	32.4 (24.77–41.17)
Missing	50	750	0.7 (0.46–1.04)
***Enabling Factors***			
**Employment status**			
Unemployed	4,064	44,222	40.8 (37.20–44.58)
Employed	5,135	63,520	58.7 (54.96–62.25)
Missing	26	553	0.5 (0.34–0.76)
**Household income (MYR)**			
MYR 0–999	5,874	70,969	65.5 (56.81–73.32)
MYR 1000 and over	3,334	36,869	34.0 (26.18–42.90)
***Health Needs Factors***			
**Recent acute health problem**			
No	7,546	87,854	81.1 (75.59–85.64)
Yes	1,644	19,898	18.4 (13.87–23.93)
Missing	35	543	0.5 (0.32–0.78)
**Self-rated health**			
Good to excellent	7,926	93,833	86.6 (82.90–89.67)
Fair, poor to very poor	1,261	13,735	12.7 (9.61–16.56)
Missing	38	727	0.7 (0.46–0.99)
**Presence of NCD**			
No NCD	7,988	93,372	86.2 (83.10–88.84)
1 NCD	770	8,920	8.2 (7.00–9.67)
2 or more NCDs	467	6,003	5.5 (4.10–7.45)
**Alcohol drinker**			
No	8,521	96,988	89.6 (85.30–92.69)
Yes	704	11,307	10.4 (7.31–14.70)
**Current tobacco user**			
No	6,177	72,676	67.1 (61.89–71.94)
Yes	2,991	34,747	32.1 (27.24–37.35)
Missing	57	872	0.8 (0.54–1.21)

Notes: %: weighted percentage; MYR: Malaysian Ringgit; NCD: non-communicable disease.

### Outpatient utilisation

Table 2 shows the overall prevalence of Orang Asli adults utilising the outpatient healthcare services in the last 12 months preceding the survey.

**Table 2:** Characteristics of adult Orang Asli users of outpatient healthcare services (n = 1,878).

Characteristics	Unweighted count	Estimated population	% (95%CI)	p-value
**Overall**	1,878	19,376	17.9 (14.83–21.43)	
***Predisposing Factors***				
**Sex**				
Male	622	5,814	12.4 (9.76–15.71)	**<0.001**
Female	1,256	13,562	22.0 (18.31–26.28)	
**Strata**				
Urban	228	336	28.2 (19.55–38.87)	0.560
Fringe	1,041	14,638	17.8 (14.19–22.11)	
Remote	609	4,402	17.7 (12.52–24.37)	
**Tribe**				
Senoi	824	12,325	21.7 (17.15–27.18)	**0.002**
Proto Malay	551	6,470	13.1 (9.92–17.08)	
Negrito	503	581	26.4 (19.05–35.24)	
**Age group (years)**				
18–39	1,095	10,316	16.1 (13.63–18.86)	**<0.001**
40–59	550	6,097	18.6 (13.80–24.55)	
60 and over	233	2,963	26.2 (22.00–30.83)	
**Marital status**				
Single/Divorced/Widowed	423	4,500	14.7 (11.64–18.42)	**<0.001**
Married	1,454	14,866	19.2 (15.70–23.38)	
Missing	1	–	–	
**Education**				
No formal education/ Never went to school	451	5,491	19.4 (16.23–23.05)	**<0.001**
Primary education (finished/attended)	778	7,310	16.6 (13.73–19.85)	
Secondary education (finished/attended)	642	6,494	18.5 (13.65–24.55)	
Missing	7	–	–	
***Enabling Factors***				
**Employment status**				
Unemployed	1,019	10,434	23.6 (20.11–27.47)	**<0.001**
Employed	856	8,880	14.0 (10.91–17.74)	
Missing	3	–	–	
**Household income (MYR)**				
MYR 0–999	1,170	12,618	17.8 (15.09–20.83)	0.675
MYR 1000 and over	707	6,741	18.3 (13.12–24.89)	
***Health Needs Factors***				
**Recent acute health problem**				
No	1,352	13,946	15.9 (13.15–19.04)	**<0.001**
Yes	524	5,402	27.2 (20.76–34.65)	
Missing	2	–	–	
**Self-rated health**				
Good to excellent	1,504	15,416	16.4 (13.33–20.08)	**<0.001**
Fair, poor to very poor	373	3,943	28.7 (23.53–34.51)	
Missing	1	–	–	
**Presence of NCD**				
No NCD	1,388	13,575	14.5 (12.27–17.14)	**<0.001**
1 NCD	270	3,058	34.3 (24.76–45.26)	
2 or more NCDs	220	2,744	45.7 (31.95–60.15)	
**Alcohol drinker**				
No	1,778	18,063	18.6 (15.38–22.37)	**0.003**
Yes	100	1,313	11.6 (9.18–14.59)	
**Smoker (any type)**				
No	1401	14947	20.6 (16.86–24.84)	**<0.001**
Yes	470	4327	12.5 (10.16–15.18)	
Missing	7	102	11.7 (5.82–22.06)	

Notes: %: weighted percentage; MYR: Malaysian Ringgit; NCD: non-communicable disease.

-: indicates relative standard error (RSE) >30.

^a^p-values are from design-based Rao-Scott chi-square tests.

The prevalence of outpatient healthcare utilisation was 17.9% (95%CI:14.83–21.43) of which females [22.0% (95%CI: 18.31–26.28)] have a higher prevalence than males [12.4% (95%CI:9.76–15.71)]. By tribes, the prevalence of outpatient healthcare utilisation among Negrito tribes is 26.4% (95%CI: 19.05–35.24), followed by Senoi [21.7% (95%CI: 17.15–27.18)], and Proto-Malay [13.1% (95%CI: 9.92–17.08)]. By locality, urban residents have a higher prevalence of outpatient use than those living in fringe and remote areas. Probing deeper into the utilisation by tribes in fringe and remote areas, the proportion of outpatient use among the Senoi tribe was found to be higher than Proto-Malay and Negrito (S1 Table). About 23.6% (95%CI: 20.11–27.47) of the Orang Asli adults were unemployed, with majority of the unemployed individuals being female (S2 Table). Additionally, adults who reported experiencing health problems in the last two weeks, self-rated their health status as fair, poor to very poor, and those diagnosed with two or more NCDs have a higher prevalence of outpatient healthcare utilisation (Table 2). Senoi adults with NCDs were found to be predominant users of outpatient services, which might contribute to the higher proportion of outpatient use by the Senoi tribe in non-urban areas (S3 Table). About 20.6% (95%CI: 16.89–24.84) and 18.6% (95%CI: 15.38–24.84) of the Orang Asli adults did not smoke any tobacco products or consume any alcoholic beverages (in the last 12 months) prior to the survey.

**Fig 2:**
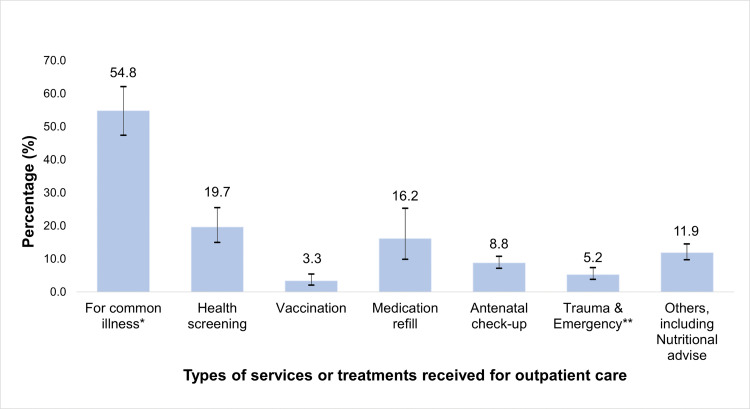
Services or treatments received for outpatient care. *Common illnesses include cough, flu, fever and muscle pain. **Trauma treatments include broken bones/open wounds/burns, etc. and emergency services for chest pain/heart attack/loss of consciousness, etc.

Additionally, the most frequent services or treatments received for outpatient care were common illnesses (54.8%), followed by health screening (19.7%), and medication refill (16.2%) (Fig 2).

### Determinants of outpatient healthcare services utilisation

After controlling for all variables, the predisposing, enabling, and needs factors contributed to outpatient healthcare utilisation were shown in Table 3.

**Table 3:** Logistic regression of determinants of outpatient healthcare services utilisation among Orang Asli adults.

Characteristics	cOR (95% CI)	p-value	aOR (95% CI)	p-value
***Predisposing Factors***				
**Sex**				
Male	Ref		Ref	
Female	1.98 (1.65–2.38)	**<0.001**	1.64 (1.31–2.06)	**<0.001**
**Strata**				
Urban	1.82 (0.97–3.42)	0.061	2.39 (1.15–4.96)	**0.020**
Fringe	1.01 (0.62–1.65)	0.964	1.13 (0.70–1.83)	0.616
Remote	Ref		Ref	
**Tribe**				
Senoi	1.83 (1.19–2.81)	**0.006**	2.66 (1.52–4.64)	**<0.001**
Proto Malay	Ref		Ref	
Negrito	2.35 (1.40–3.96)	**0.002**	3.91 (2.01–7.60)	**<0.001**
**Age group (years)**				
18–39 years	Ref		Ref	
40–59 years old	1.19 (0.96–1.46)	0.104	0.90 (0.73–1.10)	0.292
60 years old and over	1.86 (1.51–2.31)	**<0.001**	1.12 (0.88–1.43)	0.347
**Marital status**				
Single/Divorced/Widowed	Ref		Ref	
Married	1.38 (1.07–1.79)	**0.015**	1.40 (1.11–1.76)	**0.005**
**Education**				
No	Ref		Ref	
Primary education (finished/attended)	0.82 (0.66–1.03)	**0.094**	0.94 (0.74–1.19)	0.604
Secondary education (finished/attended)	0.94 (0.69–1.29)	0.710	1.13 (0.83–1.54)	0.425
***Enabling Factors***				
**Employment status**	Ref		Ref	
Unemployed	1.90 (1.60–2.27)	**<0.001**	1.27 (1.11–1.46)	**<0.001**
Employed				
**Household income (MYR)**				
MYR 0–999	Ref			
MYR 1000 and over	1.04 (0.72–1.50)	0.837		
***Health Needs Factors***				
**Recent acute health problem**				
No	Ref		Ref	
Yes	1.97 (1.52–2.55)	**<0.001**	2.17 (1.68–2.81)	**<0.001**
**Self-rated health**				
Good to excellent	Ref			
Fair, poor to very poor	2.04 (1.59–2.63)	**<0.001**	2.30 (1.39–3.79)	**0.002**
**Presence of NCD**				
No	Ref		Ref	
1 NCD	3.04 (2.03–4.57)	**<0.001**	2.90 (2.00–4.21)	**<0.001**
2 NCDs	4.91 (3.04–7.93)	**<0.001**	4.63 (2.89–7.41)	**<0.001**
**Alcohol drinker**				
No	Ref		Ref	
Yes	0.57 (0.44–0.75)	**<0.001**	1.14 (0.88–1.47)	0.306
**Current tobacco user**				
No	Ref		Ref	
Yes	0.55 (0.45–0.67)	**<0.001**	0.91 (0.76–1.11)	0.351
**Interaction**				
**Tribe x self-rated health**				
Senoi x Fair, poor to very poor			0.46 (0.24–0.87)	**<0.001**
Negrito x Fair, poor to very poor			0.30 (0.16–0.57)	**<0.001**

Note: cOR: crude odds ratio; aOR: adjusted odds ratio; CI: confidence interval; MYR: Malaysian Ringgit; NCD: non-communicable disease.

Statistically significant p-values (<0.05) are shown in bold. Archer-Lemeshow test, p = 0.092, weighted area under receiver operating characteristics (ROC) curve = 72.77% and classification table (overall correctly classified percentage = 82.73%) were applied to check the model fit. Multicollinearity was unlikely [mean VIF = 1.34 (1.03–1.81)].

The adjusted model includes all variables shown and significant interaction terms.

Among predisposing factors, females (aOR=1.64,95%CI: 1.31–2.06, p < 0.001) were more likely to utilise outpatient care in the last 12 months, as well as the Senoi (aOR=2.66,95%CI: 1.52–4.96, p < 0.05) and Negrito tribes (aOR=3.91,95%CI: 2.01–7.60, p < 0.001), and married individuals [1.40 (95%CI: 1.11–1.76, p < 0.05)]. As for enabling factors, the likelihood of using outpatient services is higher among the unemployed (aOR=1.27, 95%CI: 1.11–1.46, p < 0.05). The needs factors revealed that those who rated their health as fair, poortovery poor (aOR=2.30, 95%CI: 1.39–3.79, p < 0.05) and had one (aOR=2.90, 95%CI: 2.00–4.21, p < 0.001) or two or more NCDs (aOR=4.63, 95%CI: 2.89–7.41, p < 0.001) were associated with the likelihood of utilising outpatient healthcare services. Additionally, those experiencing recent acute health problems were also found to be significant factors influencing the outpatient use. Other covariates, such as age group and education level, alcohol and current tobacco user covariates, were not significantly associated with the outcome. Furthermore, an interaction term between tribe and self-rated health status was included in the multivariable logistic regression model. The interaction term was statistically significant [Senoi x fair, poor to very poor SRH (aOR: 0.46, 95% CI: 0.24–0.87, p < 0.001); Negrito x fair, poor to very poor SRH (aOR: 0.30, 95% CI: 0.16–0.57, p < 0.001)], indicating the odds of outpatient utilisation among Senoi or Negrito adults who rated their health as fair, poor to very poor being substantially lower than for other groups.

## Discussion

The OAHS is the first national survey that provides information on outpatient healthcare utilisation among the Orang Asli population, and the prevalence documented in this study was 17.9%. In contrast, NHMS 2023 reported the outpatient utilisation rate to be 12.5%, an increase of 8.3% from NHMS 2019 [37]. The difference in utilisation pattern among these local surveys is partly attributable to methodological differences, particularly the use of a 12-month recall period in OAHS 2022. This recall period was selected based on findings during pre-test and aligned with best practices for questionnaire structure, respondent comprehension, and overall instrument reliability and comparability.

From an international perspective, the utilisation rate amongst Orang Asli appears to be lower than that of other indigenous populations worldwide. First Nations communities in Canada reported 82.3% of adults used primary care from a healthcare practitioner in 2012, while 66% consulted a healthcare provider for non-urgent care in the past 12 months in 2024 [26,38,39]. Meanwhile, 86% of Aboriginal and Torres Strait Islander, the indigenous population in Australia, reported having seen a healthcare practitioner in the past 12 months [27]. These variations cannot be interpreted as simple utilisation gaps, as there are nuances in how outpatient care is defined. Outpatient care is delivered and financed in various mechanisms across health systems. Access pathways that range from appointment of specific primary care providers, co-payments and presence of community clinics within settlement area differ vastly amongst various indigenous communities globally [40–42]. Limited historical data on Orang Asli outpatient healthcare utilisation show limited applicability and insights to capture the intra-group differences and distinct needs of the numerous Orang Asli tribes across different geographic locations. It is imperative that Orang Asli outpatient utilisation is interpreted within its specific context to provide a meaningful interpretation that could influence policy decisions.

A scoping review of 37 studies using Andersen’s Behavioural Model identified several key predisposing factors for outpatient service use. These included female gender, marital status, older age, and unemployment [32]. Similar patterns were observed among the Orang Asli. Higher utilisation was seen among women, urban residents, unemployed individuals, and those diagnosed with NCDs. Andersen’s model focuses on how utilisation is not only influenced by individual factors but also by its enabling and environmental factors, such as service accessibility and structural characteristics of the health system. The cultural diversity and unique settlement patterns amongst the Orang Asli enable this model to be used as a lens to understand how these factors interact.

Interestingly, from our findings, the tribes of the Orang Asli also influenced service utilisation, regardless of their geographical locations. Both the Senoi and Negrito tribes, despite predominantly living in non-urban areas, had higher odds of outpatient services utilisation. On the contrary, the Proto-Malays, who were predominantly located in the urban and fringe areas, reported a lower prevalence of outpatient use. This trend indicates a potential cultural and behavioural influence on service utilisation. The gradual cultural shift towards greater acceptance of modern healthcare among Orang Asli is likely to differ among the tribe, albeit with the improved access in urban locations [19,43]. A local study among Temiar Orang Asli, a subgroup of the Senoi tribe, showcased this shift, with 50% of the study population preferring modern healthcare, while 40% preferred both modern and traditional care [19]. Further research is, however, warranted for better insights and understanding of the cultural acceptance of mainstream modern healthcare services among the Orang Asli tribes and their subgroups.

In our findings, women had a 1.64-fold higher likelihood of using outpatient services compared with men, and this is consistent with global and national trends. Women generally tend to seek healthcare more frequently due to reproductive health needs and caregiving responsibilities [44]. Gender roles, especially among indigenous females, intensify these factors, as women often serve as primary caregivers for family and community members, driving higher outpatient utilisation [42,45]. Interestingly, unemployment was also associated with higher outpatient utilisation. We noted that 88% of unemployed adults in our sample were women (S2 Table), suggesting that caregiving roles, which is commonly done by women, may contribute to more frequent engagement with health services. Benefits such as fee exemptions for Orang Asli and the availability of clinics within resettlement areas potentially minimised the financial and transportation barriers. This plausibly enhanced the greater utilisation among those not engaged in formal employment. While income was not a significant predictor in our regression model, most respondents reported household incomes of below MYR 1,000. In Malaysia, the national Poverty Line Income (PLI) in 2022 was MYR 2,589. This socioeconomic backdrop may help explain why structural supports such as fee exemptions appear to play an important role in facilitating outpatient care among unemployed women in this population.

Healthcare needs were another strong determinant of utilisation. Poor SRH and NCD diagnoses, especially multimorbidity, were major drivers of outpatient service use [40,46,47]. These findings may correspond to the growing NCD burden among the Orang Asli in Malaysia, which is potentially contributed to by resettlement and lifestyle changes [15,16,48]. Similar trends are seen among the First Nations population in Canada and indigenous populations in Australia, where chronic conditions and perceived poor health status increase primary care utilisation, thus illuminating the similar poorer health outcomes for indigenous people globally [27,39].

Another interesting finding of this study is the interaction between tribe and SRH. While poor SRH significantly predict outpatient service use in general, healthcare utilisation patterns vary among tribes. Senoi and Negrito individuals with poor health have 57–68% lower odds of healthcare use compared to their healthier counterparts. This suggests that poorer SRH does not translate to engagement in formal healthcare services, as there could be differences in interpretations of illness, thresholds to seek care or accessibility amongst these tribes. Addressing these tribe-specific health challenges requires targeted interventions to improve accessibility and preventive care for Orang Asli.

### Policy recommendations

Despite multiple initiatives to improve healthcare access for the Orang Asli communities, outpatient utilisation remains modest. Further tailoring and customisations of these initiatives to community-specific needs are crucial. Significant disease burden and the paradox of improved access not necessarily translating into equal use among tribes highlight that further improving healthcare utilisation requires more than just physical infrastructure. However, our findings showed that current targeted efforts, such as continuous strengthening and expansion of mobile health clinics and outreach services, are still warranted in certain locations and tribes. The current mobilisation efforts can be further streamlined to focus on the determinants of outpatient use among Orang Asli highlighted in this study. In resource-limited locations, focusing efforts on remote areas and in communities with greater NCD burdens can be prioritised. This also means that these efforts should be conducted hand-in-hand with screening for NCDs and ensuring the continuity of care. Digital health interventions could also be implemented, as proven by other initiatives such as digital learning to improve educational equity among Orang Asli under the 1Azam program [49].

Contrasting the higher outpatient utilisation rates and service delivery in Australia and Canada with Malaysia elucidated the presence of an indigenous-specific, community-controlled primary healthcare model. In Australia, for example, much of the primary care is provided by Aboriginal Community Controlled Health Organisations (ACCHOs), community-governed organisations funded by the government but managed by indigenous communities [50]. Similarly, in Canada, more than 89% of eligible First Nations communities are engaged in planning, management, and provision of their own health services. A detailed evaluation found that compared with state-managed or non-indigenous services, the community-controlled service achieved stronger outcomes in multidisciplinary care, community participation, culturally respectful practices, preventive and promotive health [51]. Therefore, the existing public health service deliveries in Malaysia can be further strengthened by ensuring the adoption of culturally accepted healthcare models. This may assist in further bridging modern healthcare mistrust [11,44,52].

Based on our study findings, empowering Orang Asli women could be a feasible and sustainable solution as they had high outpatient service use. They can be recruited as Orang Asli community health advocates, enabling them with simple, easily assimilated and culturally relevant health messages and information. This is similar to the successfully implemented indigenous-led initiatives in Canada [42,45]. This contextualised approach ensures Orang Asli will be able to play an active and conscious role in their own health-seeking behaviour and fosters genuine trust between Orang Asli communities and modern healthcare practitioners.

### Strength and limitations

A key strength of this study is that it is the first cross-sectional analysis that predominantly covered the Orang Asli population in Malaysia as the sample population, sharing findings that reflect healthcare utilisation patterns among this marginalised population. This provides an insight into the current utilisation trends that can inform future research and have implications for programs and policies, as Orang Asli or indigenous communities are underrepresented in nationally representative surveys or facility-based public health research. As with all studies, the limitations include the nature of the data being self-reported, which can be prone to recall bias. and underestimation, thus can limit the interpretation of study findings. The recall period for outpatient service utilisation was 12 months, differed from the standard two-week in national surveys, may cause underreporting of estimates as participants might forget their routine or low-severity outpatient visits. Another key limitation is the relatively small sample sizes for certain subgroups, particularly the Negrito and the urban population, which may limit our ability to draw definitive conclusions for these groups. Additionally, as the OAHS was cross-sectional, causal inferences about the factors influencing outpatient healthcare utilisation may be limited. Future research can incorporate longitudinal approaches to better comprehend the dynamic relationship between healthcare utilisation and health outcomes for the Orang Asli population.

## Conclusion

This study provides valuable insights into outpatient healthcare utilisation trends among the Orang Asli adults in Malaysia, revealing that 17.9% of Orang Asli adults accessed outpatient services in the last 12 months. Determinants of outpatient service utilisation include being female, living in urban areas, belonging to the Senoi or Negrito tribes, having poor self-rated health, and being diagnosed with NCDs. These findings emphasise the need for culturally sensitive interventions and policies that enhance healthcare access through strengthening and expansion of mobile clinics and outreach services, co-design and delivery of culturally accepted healthcare models and active female participation as health advocates. To improve health equity and outcomes, it is essential to optimise existing outpatient services while continually addressing persistent healthcare gaps specific to the Orang Asli population. Future research is needed to better understand the relationship between healthcare utilisation and health outcomes in this community.

## Supporting information

S1 Appendix Questionnaire and codebook from OAHS. (PDF)

S1 Table Distribution of outpatient healthcare users in the last 12 months, stratified by tribes and strata, OAHS 2022 (n = 1,878). (DOCX)

S2 Table Distribution of outpatient healthcare users in the last 12 months, stratified by sex and employment, OAHS 2022 (n = 1,878). (DOCX)

S3 Table Distribution of outpatient healthcare users in the last 12 months, stratified by tribes and non-communicable diseases, OAHS 2022 (n = 1,878). (DOCX)
