# Supplementary material for: Determinants of outpatient service use among Orang Asli in Malaysia using Andersen’s Behavioural Model
Source: PLoS One. 2026 Jan 22;21(1):e0340502. doi: 10.1371/journal.pone.0340502 (PMC12826521; doi:10.1371/journal.pone.0340502)
Supplement: S1 Appendix — (PDF) [file pone.0340502.s001.pdf]

APPENDIX 7: QUESTIONNAIRE (INDIVIDUAL)

Kementerian Kesihatan Malaysia

INSTITUTE FOR PUBLIC HEALTH @NIH

JABATAN KEMAJUAN ORANG ASLI

TINJAUAN KESIHATAN ORANG ASLI

BORANG SOAL SELIDIK
INDIVIDU

INSTITUT KESIHATAN UMUM

KEMENTERIAN KESIHATAN MALAYSIA

ISI KANDUNGAN

Bil.	Tajuk Modul		Muka Surat	Catatan
A2	Sosiodemografi	Semua umur		
B	Tingkah Laku Mendapatkan (Jagaan) Kesihatan & Penggunaan Perkhidmatan Kesihatan	Semua umur		
C	Kesihatan Mental Dewasa	≥16 tahun		
D	Penyusuan Susu Ibu & Pemakanan Bayi	0<5 tahun		
E	Morbiditi Kanak-Kanak	0<5 tahun		
F	Kesihatan Gigi Dan Mulut	≥2 tahun		
G	Kebersihan Diri	≥7 tahun		
H	Alkohol	≥13 tahun		
I	Merokok	≥13 tahun		
J	Tuberkulosis	≥15 tahun		
K	Kesihatan Wanita & Amalan Perancang Keluarga (WANITA sahaja)	15-49 tahun		
L	Kencing Manis	≥18 tahun		
M	Tekanan Darah Tinggi	≥18 tahun		
N	Paras Kolesterol Tinggi	≥18 tahun		
R	Rekod Imunisasi Kanak-Kanak	1-5 tahun		
P1	Pengukuran Antropometri	Semua umur		
P2	Anemia: Pemeriksaan Hemoglobin (WANITA sahaja)	15-49 tahun		
P3	Pemeriksaan Tekanan Darah	≥18 tahun		
P4	Pemeriksaan Glukosa Dan Kolesterol Darah	≥18 tahun		
P5	Pemeriksaan Pendedahan Kepada Logam Berat (KAMPUNG TERPILIH)	Semua umur		

Kementerian Kesihatan Malaysia

TINJAUAN KESIHATAN ORANG ASLI

INSTITUT KESIHATAN UMUM

KEMENTERIAN KESIHATAN MALAYSIA

BORANG SOAL SELIDIK (INDIVIDU)

(UNTUK DIISI OLEH PENEMURAMAH)				
ID	Negeri (2 Digit) / Daerah (2 Digit) / Kawasan (1 Digit) / Kampung (2 Digit) / TK (3 Digit) / Isirumah & Individu (IR&IND) (4 Digit)			
TARIKH TEMURAMAH	Haribulan	Bulan	Tahun	
KEPUTUSAN TEMUBUAL PERINGKAT INDIVIDU	☐ Berjaya<br>☐ Pending<br>☐ Enggan jawab<br>☐ Tiada di rumah<br>☐ Masalah bahasa<br>☐ Masalah kesihatan<br>☐ Lain-lain, nyatakan:			
KOORDINAT LOKASI GEOGRAFI	i. Latitude	☐☐☐ . ☐☐☐☐☐☐		
	ii. Longitude	☐☐☐ . ☐☐☐☐☐☐		

MODUL A2: SOSIODEMOGRAFI

A2: UNTUK DIJAWAB OLEH SETIAP AHLI ISIRUMAH

Soalan untuk diisi oleh penemuramah: Pilih SATU jawapan sahaja.

A201	Siapakah yang telah menjawab borang soal selidik ini?	1. Ahli isirumah sendiri<br>2. Ahli isirumah dibantu oleh penterjemah<br>3. Proksi (bagi pihak ahli isirumah)<br>4. Proksi dengan bantuan penterjemah
A202	Nama ahli isi rumah:	……………………………………………………………………………...
A203	Apakah jantina anda?	1. Lelaki<br>2. Perempuan
A204	Apakah hubungan anda dengan ... (nama ketua isirumah)?	1. Ketua isirumah<br>2. Suami atau isteri<br>3. Ibubapa<br>4. Anak<br>5. Datuk/ Nenek atau moyang<br>6. Cucu atau cicit<br>7. Adik-beradik<br>8. Mertua<br>9. Menantu<br>10. Ipar Duai<br>11. Saudara-mara lain<br>12. Kawan<br>13. Pekerja (pembantu rumah, tukang kebun, pemandu, lain-lain)<br>14. Lain-lain<br>(-7) TT (-9) EJ
A205	Bila tarikh lahir anda?	D D / M M / Y Y Y Y<br>**[PENEMURAMAH: Sekiranya 'TT' tuliskan '01' untuk hari, '07' untuk bulan, '9999' untuk tahun]**
A206	Berapa umur...?	[ ][ ] Tahun genap<br>(-7) TT (-9) EJ
A208	Apakah kaum anda?	1. Senoi **…sila ke A208a**<br>2. Melayu Proto **…sila ke A208b**<br>3. Negrito **…sila ke A208c**<br>(-7) TT (-9) EJ
A208a – A208c	Sila nyatakan suku kaum:<br>**[PERINGATAN: Bagi perkahwinan campur, ikut suku kaum Orang Asli; manakala bagi perkahwinan antara suku kaum Orang Asli, ikut sebelah bapa]**	**A208a:** 1. Temiar 2. Che Wong 3. Mahmeri 4. Jahut 5. Semoq Beri 6. Semai<br>**A208b:** 1. Temuan 2. Kuala 3. Kanaq 4. Seletar 5. Jakun 6. Semelai<br>**A208c:** 1. Bateq 2. Kensiu 3. Kintak 4. Lanoh 5. Jahai 6. Mendriq<br>(-7) TT (-9) EJ
A209	Apakah agama anda?<br>*Probe: Sembah apa?*	1. Islam<br>2. Buddha<br>3. Hindu<br>4. Kristian<br>5. Bahai<br>6. Agama Asal/ 'Folk'/ Animisma<br>7. Tiada agama<br>8. Lain-lain<br>(-7) TT (-9) EJ
A210	Apakah taraf perkahwinan anda?	1. Tidak pernah berkahwin …**sila ke A213**<br>2. Berkahwin<br>3. Berpisah<br>4. Janda / Duda<br>5. Balu<br>(2–5) **Jika wanita sila ke A211. Jika lelaki sila ke A213.**<br>6. Tinggal bersama pasangan<br>7. Lain-lain<br>(6–7) **sila ke A213**<br>(-7) TT (-9) EJ

A211	Berapakah umur anda ketika berkahwin (**perkahwinan pertama**)?<br><br>*Probe: Anak sulung umur berapa sekarang?*	Umur: ☐☐ Tahun<br>(-7) TT (-9) EJ
A212	Berapakah umur pasangan anda ketika berkahwin (**perkahwinan pertama**)?<br><br>*Probe: suami umur berapa sekarang?*	Umur: ☐☐ Tahun<br>(-7) TT (-9) EJ
A213	Apakah tahap pendidikan **tertinggi** anda?	1. Tidak pernah bersekolah<br>2. Tidak habis sekolah rendah<br>3. Tamat darjah 6<br>4. Tamat tingkatan 3<br>5. Tamat tingkatan 5<br>6. Tamat tingkatan 6/ sijil/ diploma<br>7. Tamat pengajian peringkat sarjana muda atau tahap lebih tinggi<br>8. Pendidikan secara tidak formal (contohnya: sekolah pondok, sekolah mubaligh, sekolah komuniti)<br>(-7) TT (-9) EJ
A214	Dalam **tempoh 1 bulan lepas**, adakah anda bekerja untuk mendapatkan duit?	1. Ya …**sila keA216**<br>2. Tidak<br>(-7) TT (-9) EJ<br>**…sila ke A217 jika TT/ EJ**
A215	Jika tidak, kenapa?<br><br>**[Pilih SATU jawapan UTAMA sahaja.]**	1. Masalah kesihatan/kurang upaya<br>2. Menjaga pesakit/orang kurang upaya/orang tua<br>3. Menjaga rumah/anak-anak, cucu, ahli keluarga lain<br>4. Menganggur<br>5. Pelajar<br>6. Pesara<br>7. Tua<br>8. Kanak-kanak tidak bersekolah/ belum bersekolah<br>9. Faktor cuaca/ musim/ bencana alam<br>10. Tiada peluang bekerja bergaji<br>11. Lain-lain<br>(-7) TT (-9) EJ<br>**Terus ke A217 selepas soalan ini**
A216	Apakah pekerjaan utama anda?<br><br>**[Pilih SATU jawapan UTAMA sahaja.]**	1. Pengurus<br>2. Profesional<br>3. Juruteknik dan professional bersekutu<br>4. Pekerja sokongan perkeranian<br>5. Pekerja perkhidmatan dan jualan<br>6. Pekerja mahir pertanian, perhutanan, penternakan, dan perikanan<br>7. Pekerja kemahiran dan pekerja pertukangan yang berkaitan<br>8. Operator mesin dan loji, dan pemasang<br>9. Pekerja asas<br>10. Angkatan tentera<br>(-7) TT (-9) EJ
Berapakah purata pendapatan kasar anda sebulan, dari segi ...<br><br>**Sekiranya TIADA pendapatan tuliskan '0'**		
A217	…pendapatan bulanan tetap dari bekerja / gaji / upah / pencen	RM ☐☐☐☐☐☐☐ . ☐☐ Sebulan<br>(-7) TT (-9) EJ
A218	…wang yang diterima daripada ahli isirumah lain	RM ☐☐☐☐☐☐☐ . ☐☐ Sebulan<br>(-7) TT (-9) EJ
A219	...wang daripada sumber lain, contohnya daripada jualan hasil hutan/ pertanian/ penternakan/ sumber semula jadi	RM ☐☐☐☐☐☐☐ . ☐☐ Sebulan<br>(-7) TT (-9) EJ
A220	...wang daripada sumber lain, contohnya wang daripada ahli keluarga di luar isirumah, biasiswa, kebajikan masyarakat//sosial, kutipan sewa aset, Baitulmal, dividen dan lain-lain	RM ☐☐☐☐☐☐☐ . ☐☐ Sebulan<br>(-7) TT (-9) EJ

MODUL B: TINGKAH LAKU MENDAPATKAN (JAGAAN) KESIHATAN & PENGGUNAAN PERKHIDMATAN KESIHATAN

KRITERIA KELAYAKAN: SETIAP AHLI ISIRUMAH

Soalan-soalan berikut berkenaan tingkahlaku mendapatkan (jagaan) kesihatan & penggunaan perkhidmatan kesihatan.

B000	Adakah anda berumur 13 tahun dan keatas?	1. Ya<br>2. Tidak ...**sila ke B200**
B100	Bagaimanakah anda menilai tahap kesihatan anda?<br><br>*Probe: Sesihat mana anda rasa sekarang?*	1. Sangat bagus<br>2. Bagus<br>3. Sederhana<br>4. Tidak bagus<br>5. Sangat tidak bagus<br>(-7) TT (-9) EJ
**[PENEMURAMAH: Soalan-soalan berikutnya adalah untuk semua umur. Sila rujuk Buku Kod untuk soalan-soalan yang merujuk kepada Kod A dan Kod B.]**		
B200	Dalam tempoh 2 minggu lepas, adakah anda mengalami mana-mana masalah ini? (Bacakan senarai pada Kod A)<br><br>**[PENEMURAMAH: Sila bacakan pilihan senarai pada Kod A]**	1. Ya<br>2. Tidak …**sila ke B300**<br>(-7) TT (-9) EJ<br>**…sila ke B300 jika TT/EJ**
B201	Bagi …[masalah yang disebutkan pada B200]…, adakah anda **mengubati diri sendiri**?	1. Ya, rawatan tradisional<br>2. Ya, makan ubat klinik/hospital (rawatan moden)<br>3. Ya, kedua-dua rawatan tradisional dan Moden<br>4. Tidak<br>(-7) TT (-9) EJ
B202	Bagi …[masalah yang disebutkan pada B200]…, adakah anda **mendapatkan nasihat** dan/atau rawatan daripada mana-mana **klinik/hospital (termasuk klinik bergerak)**?	1. Ya<br>2. Tidak<br>(-7) TT (-9) EJ
B203	Bagi …[masalah yang disebutkan pada B200]…, adakah anda **membeli ubat dari farmasi selepas dinasihatkan** oleh mana-mana **ahli farmasi**?	1. Ya<br>2. Tidak<br>(-7) TT (-9) EJ
B204	Bagi …[masalah yang disebutkan pada B200]…, adakah anda **mendapatkan rawatan tradisional daripada tok halak/bomoh/dukun/pawang?**	1. Ya<br>2. Tidak<br>(-7) TT (-9) EJ
B300	Bilakah **kali terakhir anda pergi ke klinik/hospital** (termasuk klinik bergerak)? (Nota: Tidak termasuk rawatan pergigian.)<br><br>**[PENEMURAMAH/SISTEM: Soalan wajib dijawab.]**	1. Dalam masa satu tahun lepas<br>2. Lebih dari satu tahun lepas<br>3. Tidak pernah<br>(-7) TT (-9) EJ
B301	Dalam tempoh 12 bulan terakhir, adakah **anda merasa perlu** rawatan di klinik/hospital (termasuk klinik bergerak) tetapi **anda tidak menerima-/mendapatkannya**?<br><br>*Probe: Ada rasa nak pergi tapi tak pergi?*<br><br>**[PENEMURAMAH/SISTEM: Soalan wajib dijawab.]**	1. Ya<br>2. Tidak …**sila ke B400**<br>(-7) TT (-9) EJ
B302	Apakah **sebab utama** anda **tidak mendapatkan rawatan/ubat atau nasihat** daripada klinik/hospital (termasuk klinik bergerak)?<br><br>*Probe: Kenapa tak pergi?*<br><br>**[PENEMURAMAH: Pilih SATU jawapan sahaja. Sekiranya responden sakit berulang-kali, mohon nyatakan untuk keadaan kesakitan yang paling teruk sahaja.]**	1. Tidak mampu bayar untuk rawatan/ kos pengangkutan<br>2. Tiada pengangkutan untuk pergi<br>3. Sangat tidak sihat untuk pergi<br>4. Tiada lawatan pasukan bergerak<br>5. Tidak berada di kampung semasa pasukan bergerak datang<br>6. Tidak tahu tempat mana hendak pergi<br>7. Takut ke klinik/hospital (takut jumpa doktor)<br>8. Pernah dilayan dengan buruk sebelum ini<br>9. Anda cuba pergi, tapi tidak diterima<br>10. Anda rasa sakit ringan sahaja (tidak penting)<br>11. Anda memilih perubatan tradisional berbanding perubatan moden<br>12. Anda mengikut pendapat/nasihat tok halak/bomoh/pawang/dukun<br>13. Masalah bahasa<br>(-7) TT (-9) EJ
B400	Dalam tempoh 12 bulan terakhir, adakah anda **pergi ke klinik/hospital/klinik bergerak tetapi tidak bermalam** (jagaan kesihatan pesakit luar)?<br><br>*Probe: Terus balik atau tidur situ? Takde kena tidur malam kan?*<br><br>**[PENEMURAMAH: Jagaan ini TIDAK MEMERLUKAN responden untuk bermalam dan tidak melibatkan jagaan kesihatan mulut atau gigi.]**<br><br>**[PENEMURAMAH/SISTEM: Soalan wajib dijawab.]**	1. Ya<br>2. Tidak …**sila ke B500**<br>(-7) TT (-9) EJ<br>**…sila ke B500 jika TT/EJ**

[PENEMURAMAH: Isikan bagi SATU tempat TERKINI untuk B411-B433]		
B411	Di manakah tempat anda mendapatkan perkhidmatan kesihatan tersebut?	1. Klinik dengan bangunan(Klinik kesihatan, klinik desa)<br>2. Hospital<br>3. Klinik bergerak (darat/air/udara)<br>(-7) TT (-9) EJ
B412	Adakah tempat tersebut milik kerajaan atau swasta?	1. Kerajaan<br>2. Swasta<br>(-7) TT (-9) EJ
B413	Apakah perkhidmatan/rawatan yang anda terima?<br>(Nota: Tidak termasuk rawatan pergigian.)<br><br>**[PENEMURAMAH: Sila bacakan pilihan jawapan. Lebih dari satu jawapan diterima untuk soalan ini (kecuali jika TT dan EJ).]**	1. Untuk sakit biasa (rujuk Kod A)<br>2. Jagaan antenatal (ibu mengandung)<br>3. Vaksinasi<br>4. Isi ulang ubat<br>5. Trauma (patah tulang/putus jari/luka bakar/luka tusuk/luka yang sangat besar)<br>6. Kecemasan (serangan jantung/sesak nafas/hilang kesedaran/muntah darah)<br>7. Saringan kesihatan (periksa tekanan darah/kadar gula)<br>8. Nasihat pemakanan<br>9. Lain-lain<br>(-7) TT (-9) EJ
B420	Siapakah yang bayar rawatan?	1. Pengecualian bayaran/tidak bayar apa-apa/ "*tunjuk surat*"<br>2. Skim PeKa B40<br>3. Insurans kesihatan peribadi (swasta)<br>4. Majikan dan/atau klinik/hospital panel<br>5. Bayar sendiri<br>6. Lain-lain<br>(-7) TT (-9) EJ
B431	Apakah **jenis pengangkutan** yang anda guna untuk ke [jawapan pada B411]...?<br><br>**[PENEMURAMAH: Sila bacakan pilihan jawapan. Lebih dari satu jawapan diterima untuk soalan ini (kecuali jika TT dan EJ).]**	1. Kereta sendiri/ tumpang/pinjam<br>2. Motosikal/basikal sendiri/ tumpang/pinjam<br>3. Bot/perahu sendiri/ tumpang/pinjam<br>4. Kenderaan (kereta/bot) jabatan<br>5. Kenderaan sewa atau awam<br>6. Jalan kaki<br>7. Ambulans<br>8. Lain-lain<br>(-7) TT (-9) EJ
B432	Biasanya berapa lama **masa perjalanan sehala** dari rumah ke ...[jawapan pada B411]...?<br><br>**[PENEMURAMAH: Jangan tinggalkan ruangan kosong. Sila isikan '0' jika tidak berkenaan. Contoh: 30 minit -> 0 hari 0 jam 30 minit.]**	☐ Jam ☐ Hari ☐ Minit<br>(-7) TT (-9) EJ
B433	Berapakah **anggaran jarak** dari rumah anda ke ...[jawapan pada B411]...?	.... km<br>(-7) TT (-9) EJ
B500	Dalam tempoh 12 bulan terakhir, adakah anda **dimasukkan ke wad** di hospital?<br><br>**[PENEMURAMAH: Responden dikira sebagai bermalam sekiranya kemasukan ke wad di hospital melepasi 12 tengah malam. Ini TIDAK TERMASUK seseorang yang dimasukkan ke tempat rawatan dan discaj pada hari yang sama, lawatan ke klinik pesakit luar, rawatan yang tidak berkaitan dengan kesihatan, menemani pesakit bermalam sebagai ahli keluarga atau dimasukkan ke pusat rawatan harian (daycare)].**<br><br>**[PENEMURAMAH/SISTEM: Soalan wajib dijawab.]**	1. Ya<br>2. Tidak …**sila ke B600**<br>(-7) TT (-9) EJ<br>**…sila ke B600 jika TT/EJ**
B501	Apakah **sebab utama** anda dimasukkan ke wad di hospital?<br>(Bacakan/rujuk senarai pada Kod B)<br><br>**[PENEMURAMAH: Sila bacakan pilihan jawapan pada Kod B. Isikan nombor sahaja.]**	……………………………………………….<br>(-7) TT (-9) EJ
B600	Siapa yang **biasanya tentukan** samada **anda perlu pergi ke klinik/hospital** (termasuk klinik bergerak)**?**<br><br>**[PENEMURAMAH: Pilih SATU jawapan sahaja]**<br><br>**[PENEMURAMAH/SISTEM: Soalan wajib dijawab.]**	1. Suami atau isteri atau pasangan<br>2. Ibu atau bapa<br>3. Anak<br>4. Ahli keluarga lain<br>5. Sendiri<br>6. Penjawat awam<br>7. Tok Halak/Bomoh/Dukun/Pawang<br>8. Tok Batin/Ketua Kampung/Ketua Masyarakat<br>9. Lain-lain<br>(-7) TT (-9) EJ
**Tamat, ke Modul seterusnya**		

MODUL C: KESIHATAN MENTAL DEWASA

KRITERIA KELAYAKAN: AHLI ISIRUMAH UMUR 16 TAHUN DAN KE ATAS

Pihak kami ingin mengetahui samada anda mempunyai sebarang masalah perubatan, dan bagaimana kesihatan anda secara umum, sejak **beberapa minggu kebelakangan ini (kurang dari sebulan)**. Sila jawab **semua soalan** dan pilih jawapan yang paling hampir dengan keadaaan anda sekarang. **SILA AMBIL PERHATIAN** yang kami ingin mengetahui masalah terkini, dan bukannnya masalah di masa lalu. Adalah sangat penting untuk anda menjawab **SEMUA** soalan di bawah.

Sila tandakan [√] Terima kasih.

PERNAHKAH BARU-BARU INI ANDA:

1.	Boleh menumpukan perhatian kepada apa sahaja yang dibuat?<br>*Probe: semasa melakukan sesuatu kerja, dapat tak melakukannya hingga siap*	☐ Lebih baik dari biasa	☐ Sama seperti biasa	☐ Kurang dari biasa	☐ Sangat kurang dari biasa
2.	Kekurangan tidur kerana risau?<br>*Probe: tidak dapat tidur sebab banyak fikir benda*	☐ Tidak sama sekali	☐ Tidak lebih dari biasa	☐ Agak lebih dari biasa	☐ Sangat lebih dari biasa
3.	Rasa yang anda memainkan peranan yang berguna dalam banyak perkara?<br>*Probe: contoh seorang ibu, dapat menjaga anak dengan baik, seorang bapa dapat mencari duit/makanan untuk keluarga, seorang anak dapat membantu ibu/bapa dengan baik*	☐ Lebih dari biasa	☐ Sama seperti biasa	☐ Kurang dari biasa	☐ Sangat kurang dari biasa
4.	Merasa mampu membuat keputusan tentang sesuatu?<br>*Probe: rasa dapat buat keputusan*<br>*Contoh: apa yang ingin dimasak (ibu & anak perempuan), mana mahu cari duit/makanan (bapa & anak lelaki)*	☐ Lebih dari biasa	☐ Sama seperti biasa	☐ Kurang dari biasa	☐ Sangat kurang mampu
5.	Sentiasa merasa tertekan / tegang?<br>*Probe: selalu merasa stress*	☐ Tidak sama sekali	☐ Tidak lebih dari biasa	☐ Agak lebih dari biasa	☐ Sangat lebih dari biasa
6.	Rasa yang tidak dapat mengatasi kesukaran / masalah anda?<br>*Probe: jika ada sesuatu masalah, rasa/fikir tidak dapat selesaikannya*	☐ Tidak sama sekali	☐ Tidak lebih dari biasa	☐ Agak lebih dari biasa	☐ Sangat lebih dari biasa
7.	Dapat menikmati kegiatan harian anda?<br>*Probe: rasa gembira/seronok/enjoy buat kerja harian*	☐ Lebih dari biasa	☐ Sama seperti biasa	☐ Kurang dari biasa	☐ Sangat kurang dari biasa
8.	Dapat mengatasi masalah-masalah anda?<br>*Probe: jika ada masalah, dapat selesaikannya*	☐ Lebih dari biasa	☐ Sama seperti biasa	☐ Kurang dari biasa	☐ Sangat kurang dari biasa
9.	Merasa tidak gembira dan sedih?<br>*Probe: rasa tidak happy/ tidak seronok/ selalu menangis*	☐ Tidak sama sekali	☐ Tidak lebih dari biasa	☐ Agak lebih dari biasa	☐ Sangat lebih dari biasa
10.	Telah hilang kepercayaan pada diri anda sendiri?<br>*Probe: rasa tidak yakin diri sendiri*<br>*Contoh: rasa tidak dapat masak dengan baik (ibu & anak perempuan), rasa tidak dapat cari duit/makanan untuk keluarga (bapa &anak lelaki)*	☐ Tidak sama sekali	☐ Tidak lebih dari biasa	☐ Agak lebih dari biasa	☐ Sangat lebih dari biasa
11.	Memikirkan diri anda seorang yang tidak berguna?<br>*Probe: fikir tidak ada orang yang ambil tahu tentang hal anda/ fikir tiada orang mahu berkawan dengan anda*<br>*Contohnya fikir kalau tidak ada pun, tiada orang kisah*	☐ Tidak sama sekali	☐ Tidak lebih dari biasa	☐ Agak lebih dari biasa	☐ Sangat lebih dari biasa
12.	Rasa cukup gembira dalam segala hal yang difikirkan?<br>*Probe: seronok dalam semua perkara (keluarga/kawan-kawan/tanaman/haiwan ternakan)*	☐ Lebih baik dari biasa	☐ Sama seperti biasa	☐ Kurang dari biasa	☐ Sangat kurang dari biasa

MODUL D: PENYUSUAN SUSU IBU & PEMAKANAN BAYI		
**KRITERIA KELAYAKAN: KANAK-KANAK UMUR 0 – 59 BULAN**		
D101	Adakah (nama anak) pernah disusukan susu ibu (susu badan)?	1. Ya<br>2. Tidak …**sila ke D105**<br>(-7) TT (-9) EJ
D102	Adakah anak masih disusukan dengan susu ibu (susu badan) sahaja?	1. Ya<br>2. Tidak<br>(-7) TT (-9) EJ
D103	Adakah anak masih disusukan dengan susu ibu di samping diberikan makanan/minuman yang lain?	1. Ya …**sila ke D105**<br>2. Tidak<br>(-7) TT (-9) EJ
D104	Berapa umur anak semasa berhenti menyusu susu ibu (susu badan)?	☐☐ Bulan<br>(-7) TT (-9) EJ
D105	Berapa umur anak mula diberikan makanan/ minuman (*complementary feeding*)?	☐☐ Bulan<br>(-7) TT (-9) EJ

MODUL E: MORBIDITI KANAK-KANAK		
**KRITERIA KELAYAKAN: KANAK-KANAK UMUR 0 – 59 BULAN**		
E100	Dalam masa **2 minggu yang lepas**, adakah (nama anak) pernah ada cirit-birit?<br>*Cirit-birit = tiga atau lebih najis berair sehari, atau darah dalam najis seperti yang dilihat oleh ibu/penjaga	1. Ya<br>2. Tidak …**sila ke E103**<br>(-7) TT (-9) EJ<br>**…sila ke E103 jika TT/EJ**
E101	Sewaktu cirit-birit itu, adakah……. (nama anak) diberikan cecair daripada pek khas bernama ORS (air garam) untuk diminum?	1. Ya<br>2. Tidak<br>(-7) TT (-9) EJ
E102	Adakah antibiotik diberikan untuk merawat cirit-birit tersebut?	1. Ya<br>2. Tidak<br>(-7) TT (-9) EJ
E103	Biasanya, setelah…. (nama anak) membuang air besar, apakah yang dilakukan untuk membuang najis tersebut?	1. Kanak-kanak menggunakan tandas/ jamban<br>2. Diletakkan/ dicuci ke dalam tandas atau Jamban<br>3. Dibuang/ dicuci ke dalam longkang atau parit/ sungai/ laut<br>4. Dibuang ke dalam sampah (sisa pepejal)<br>5. Ditanam<br>6. Dibiarkan secara terbuka<br>7. Jawapan selain di atas<br>(-7) TT (-9) EJ
E104	Dalam **2 minggu yang lepas**, pernahkah (nama anak) mengalami ………	
	a. demam?	1. Ya<br>2. Tidak<br>(-7) TT (-9) EJ
	b. batuk?	1. Ya<br>2. Tidak<br>(-7) TT (-9) EJ
	c. pernafasan laju atau susah bernafas?	1. Ya<br>2. Tidak<br>(-7) TT (-9) EJ
**Jika 'Ya' kepada mana-mana di E104, jawab soalan seterusnya.<br>Jika 'Tidak/ TT/ EJ' untuk E104a hingga E104c, tamat soalan dan ke Modul seterusnya.**		
E105	a. Adakah anda mendapatkan nasihat atau rawatan?	1. Ya<br>2. Tidak …**tamat, ke Modul seterusnya**<br>(-7) TT (-9) EJ<br>**...tamat modul jika TT/EJ**
	b. Daripada mana anda mendapatkan nasihat atau rawatan?<br>**[Boleh pilih lebih dari satu jawapan]**	1. Fasiliti atau pengamal kesihatan kerajaan/<br>2. Fasiliti atau pengamal kesihatan swasta<br>3. Tempat pengamal kesihatan tradisional atau komplementari seperti Tok Halak, sinseh, bomoh<br>4. Lain-lain seperti farmasi, kedai, atau jiran<br>(-7) TT (-9) EJ
	c. Adakah (nama anak) dimasukkan ke hospital bagi kejadian ini? (≥24 JAM)	1. Dimasukkan ke wad<br>2. Tidak masuk wad<br>(-7) TT (-9) EJ
E106	Adakah (nama anak) diberikan antibiotik untuk merawat sakit ini?	1. Ya<br>3. Tidak<br>(-7) TT (-9) EJ
**Tamat, ke Modul seterusnya**		

MODUL F: KESIHATAN GIGI DAN MULUT

KRITERIA KELAYAKAN: AHLI ISIRUMAH UMUR 2 TAHUN DAN KE ATAS

Bagi setiap perkara, sila bulatkan pilihan jawapan yang paling berkaitan dengan anda.

**F1. Soalan yang berikut bertanyakan mengenai amalan seharian.**		
F101	Pada kebiasaannya, berapa kali dalam sehari anda membersih atau menggosok gigi anda/ anak anda?<br><br>**[Sila tunjuk kad imbasan]**	1. Tidak menggosok gigi …**sila ke F201**<br>2. Sekali Sekala<br>3. 1 kali sehari<br>4. 2 kali sehari<br>5. 3 kali atau lebih sehari<br>(-7) TT (-9) EJ
F102	Pada kebiasaannya, bagaimana anda membersihkan gigi atau menggosok gigi anda/ anak anda<br><br>**[Sila tunjuk kad imbasan]**	1. Menggosok gigi tanpa ubat gigi<br>2. Menggosok gigi dengan ubat gigi<br>3. Cara Tradisional<br>4. Tidak menggosok gigi<br>(-7) TT (-9) EJ
**F2. Soalan yang berikut bertanyakan mengenai kemudahan fasiliti pergigian.**		
F201	Dalam masa **12 bulan lepas**, adakah anda/ anak anda berjumpa doktor gigi/ misi gigi?<br><br>**[Sila tunjuk kad imbasan]**	1. Ya<br>2. Tidak …**soalan tamat bagi responden umur 12 tahun ke bawah**<br>(-7) TT (-9) EJ
F202	Dimanakah tempat anda/ anak anda berjumpa doktor gigi/ misi gigi?<br><br>**[Pilih jawapan yang paling kerap dikunjungi]**	1. Klinik Pergigian Kerajaan<br>2. Klinik Pergigian Swasta<br>3. Tukang gigi atau kedai gigi<br>4. Pasukan Pergigian Bergerak<br>5. Kem Kesihatan<br>6. Lain-lain<br>(-7) TT (-9) EJ
F203	Apakah sebab anda/ anak anda berjumpa doktor gigi/ misi gigi?<br><br>**[Boleh pilih lebih dari satu jawapan]**	1. Pemeriksaan mulut dan gigi<br>2. Pembersihan gigi / Cuci gigi<br>3. Sakit gigi / Gigi berlubang<br>4. Gigi goyang<br>5. Rasa ngilu/ sensitif<br>6. Gusi bengkak, berdarah atau bernanah<br>7. Gigi tercabut atau kehilangan gigi<br>8. Gigi palsu<br>9. Rawatan gigi seperti tampalan / cabutan<br>10. Rawatan gigi tidak teratur<br>11. Rawatan pecah mulut atau mata ikan<br>12. Pemeriksaan atau rawatan kanser mulut atau gigi.<br>(-7) TT (-9) EJ
**Soalan seterusnya hanya untuk 13 tahun keatas sahaja.**		
**F3. Soalan yang berikut bertanyakan mengenai maklumat penjagaan gigi.**		
F301	Pernahkah anda mendapat nasihat/maklumat mengenai penjagaan gigi?<br><br>**[Sila tunjuk kad imbasan]**	1. Pernah<br>2. Tidak pernah …**sila ke F401**<br>(-7) TT (-9) EJ<br>**…sila ke F401 jika TT/EJ**
F302	Bagaimana anda mendapat nasihat/maklumat mengenai penjagaan gigi?<br><br>**[Boleh pilih lebih dari satu jawapan]**<br>**[Sila tunjuk kad imbasan]**	1. Nasihat dari doktor gigi/ misi gigi<br>2. Keluarga<br>3. Tok Halak/ Tok Batin/ Tok Bomoh/ Dukun<br>4. Media sosial (Televisyen/ Radio/ Internet)<br>5. Kem/Pameran Kesihatan Pergigian<br>6. Pasukan Kesihatan Sekolah<br>7. Lain-lain<br>(-7) TT (-9) EJ
**F4. Soalan yang berikut bertanyakan mengenai amalan mengunyah daun sirih.**		
F401	Pada kebiasaannya, adakah anda mengamalkan mengunyah daun sirih dan/atau buah pinang?<br><br>**[Sila tunjuk kad imbasan]**	1. Ya …**sila ke F402**<br>2. Tidak …**tamat, ke Modul seterusnya**<br>(-7) TT (-9) EJ<br>**…sila ke F402 jika TT/EJ**
F402	Jenis kunyahan daun sirih yang selalu diamalkan?<br><br>**[Sila tunjuk kad imbasan]**	1. Kunyahan daun sirih<br>2. Kunyahan daun sirih Bersama tembakau<br>3. Kunyahan buah pinang sahaja<br>(-7) TT (-9) EJ

Definisi kunyahan daun sirih:
Kunyahan daun sirih ialah kunyahan daun sirih bersama kapur sirih (jika ada) dan buah pinang.

Tamat, ke Modul seterusnya

MODUL G: KEBERSIHAN DIRI		
**KRITERIA KELAYAKAN: AHLI ISIRUMAH BERUMUR 7 TAHUN DAN KE ATAS**		
G101	Pada kebiasaannya, adakah anda menggunakan tandas di kawasan rumah anda?<br><br>**[PENEMURAMAH: Sila tanya dan memerhatikan keadaan tandas sebenar jika jawab "Ya"]**	1. Ya …**sila ke G104**<br>2. Tidak<br>3. Tiada tandas …**sila ke G105**<br><br>(-7) TT (-9) EJ<br>**…sila ke G105 jika TT/EJ**
G102	Jika "Tidak", kenapa anda tidak menggunakan tandas tersebut?<br><br>**[Boleh pilih lebih daripada satu jawapan]**	1. Tak suka tandas di dalam rumah<br>2. Tiada air<br>3. Pengudaraan tidak mencukupi<br>4. Guna untuk penyimpanan barang<br>5. Tidak suka menggunakan tandas<br>6. Kepercayaan adat<br>7. Kerana tandas berbumbung<br>8. Tandas rosak<br>9. Menggunakan tandas komuniti<br>(2–9: **Sila ke G105**)<br><br>(-7) TT (-9) EJ
G103	Kenapa anda tidak suka tandas di dalam rumah?<br><br>**[Boleh pilih lebih daripada satu jawapan]**	1. Tiada air<br>2. Suka terbuka<br>3. Amalan / budaya<br>4. Berbau<br><br>(-7) TT (-9) EJ<br>**…sila ke G105 jika TT/EJ**
G104	Adakah anda menggunakan tandas <u>setiap kali</u> anda membuang air besar?	1. Ya<br>2. Kadang-kadang<br>3. Tidak<br><br>(-7) TT (-9) EJ
G105	Pada kebiasaannya, adakah anda mencuci tangan selepas buang air besar?	1. Ya<br>2. Tidak …**sila ke G107**<br><br>(-7) TT (-9) EJ
G106	Jika "Ya", pada kebiasaannya, apakah yang anda gunakan semasa mencuci tangan?	1. Air sahaja<br>2. Air dan sabun<br>3. Lain-lain<br><br>(-7) TT (-9) EJ
G107	Pada kebiasaannya, adakah anda mencuci tangan sebelum makan?	1. Ya<br>2. Tidak<br><br>(-7) TT (-9) EJ
G108	Berapa kerap anda mandi?	1. Setiap hari<br>2. 2-3 hari sekali<br>3. Seminggu sekali atau lebih<br><br>(-7) TT (-9) EJ
G109	Status kebersihan kuku:<br><br>**[Pemerhatian oleh penemuramah]**	1. Bersih<br>2. Tidak bersih<br><br>(-9) EJ
G110	Pada kebiasaannya, adakah anda memakai alas kaki (kasut/selipar/but) apabila keluar rumah?	1. Sentiasa<br>2. Kadang-kadang<br>3. Tidak<br><br>(-7) TT (-9) EJ
**Tamat, ke Modul seterusnya**		

MODUL H: ALKOHOL

KRITERIA KELAYAKAN: AHLI ISIRUMAH BERUMUR 13 TAHUN DAN KE ATAS

ARAHAN

1. Kertas soal selidik ini mengandungi 13 soalan untuk dijawab sendiri dengan bantuan penemuramah oleh responden yang berumur **13 tahun dan ke atas. SEMUA JAWAPAN ADALAH SULIT.**
2. Untuk jawapan yang dipilih, sila tandakan (✓) di kotak yang disediakan. Sila pilih **SATU JAWAPAN** sahaja.
3. Bagi sesetengah soalan, anda mungkin perlu melompat ke soalan yang tertentu berdasarkan jawapan yang dipilih.
   - Untuk soalan H104, tamat modul ini jika soalan ini dijawab sebagai "Tidak Pernah".
   - Untuk soalan H105 dan H106, sila teruskan ke soalan H112 jika soalan H105 dijawab sebagai "1 atau 2" dan soalan H106 dijawab sebagai "Tidak Pernah".
   - Untuk H114, tamat modul jika soalan ini dijawab sebagai "Tidak/TT/EJ"
   - Jika tiada arahan khusus bagi sesuatu jawapan yang dipilih, anda dikehendaki menjawab soalan yang seterusnya (mengikut turutan nombor).

Jika anda mempunyai sebarang kemusykilan semasa menjawab, sila dapatkan bantuan daripada penemuramah.

H101	Pernahkah anda minum sebarang minuman yang memabukkan? (seperti engkem, arak, bir, wain, minuman herba berarak, todi, samsu, tuak, whisky, stout dan lain-lain)<br><br>1. Ya<br>2. Tidak …**tamat, sila ke Modul seterusnya**
H102	Dalam masa **12 bulan yang lepas**, adakah anda minum sebarang minuman yang memabukkan? (seperti engkem, arak, bir, wain, minuman herba berarak, todi, samsu, tuak, whisky, stout dan lain-lain)<br><br>1. Ya<br>2. Tidak

Sila tandakan (✓) pada kotak yang bersesuaian.

H103	Apakah jenis minuman yang memabukkan yang paling kerap anda minum?	Sila tandakan (✓) **sekali sahaja** iaitu pada minuman yang **paling kerap** diminum.<br><br>☐ Shandy<br>☐ Bir ATAU Lager ATAU Ale ATAU Stout<br>☐ Wain ATAU Cider ATAU Champagne ATAU Peri ATAU Todi<br>☐ Tuak ATAU Tuak Kelapa ATAU Bahar ATAU Lihing ATAU Ijok<br>☐ Brandi ATAU Rum ATAU Wiski ATAU Vodka ATAU Gin ATAU Samsu ATAU Samsu Cheng<br>☐ Montoku ATAU Langka<br>☐ Lain-lain<br>☐ Tidak Tahu
H104	Dalam tempoh **12 bulan yang lepas**, berapa kerapkah anda minum minuman yang memabukkan?	☐ Tidak Pernah...**Tamat modul**<br>☐ Sekali sebulan atau kurang<br>☐ 2-4 kali sebulan<br>☐ 2-3 kali seminggu<br>☐ 4 kali atau lebih seminggu
H105	Kebiasaanya pada hari yang anda minum, berapa banyakkah anda minum minuman yang memabukkan?<br><br>Jumlah pengambilan minuman yang memabukkan mestilah mengikut unit minuman beralkohol seperti yang ditunjukkan dalam **Kad Imbasan**.	☐ 1 atau 2<br>☐ 3 atau 4<br>☐ 5 atau 6<br>☐ 7, 8 atau 9<br>☐ 10 atau lebih<br>☐ Tidak Tahu<br>☐ Enggan Jawab
H106	Berapa kerap anda minum enam unit atau lebih minuman yang memabukkan pada satu masa?	☐ Tidak Pernah<br>☐ Kurang dari sekali sebulan<br>☐ Sekali sebulan<br>☐ Sekali seminggu<br>☐ Setiap hari atau hampir setiap hari<br>☐ Tidak Tahu<br>☐ Enggan Jawab

JIKA SOALAN H105 DIJAWAB SEBAGAI "1 ATAU 2" DAN SOALAN H106 DIJAWAB SEBAGAI "**TIDAK PERNAH**" TERUS KE SOALAN H112.			
H107	Dalam tempoh **12 bulan yang lepas**, berapa kerapkah anda tidak boleh berhenti minum apabila anda mula minum minuman yang memabukkan?<br><br>*Probe: tidak boleh berhenti minum engkem setelah mula minum*	☐ Tidak Pernah<br>☐ Kurang dari sekali sebulan<br>☐ Sekali sebulan	☐ Sekali seminggu<br>☐ Setiap hari atau hampir setiap hari<br>☐ Tidak Tahu<br>☐ Enggan Jawab
H108	Dalam tempoh **12 bulan yang lepas**, akibat dari minum minuman yang memabukkan, berapa kerapkah anda tidak boleh melakukan apa yang biasanya anda lakukan?<br>*Probe: tidak dapat melakukan kerja yang anda biasa buat akibat minum engkem*	☐ Tidak Pernah<br>☐ Kurang dari sekali sebulan<br>☐ Sekali sebulan	☐ Sekali seminggu<br>☐ Setiap hari atau hampir setiap hari<br>☐ Tidak Tahu<br>☐ Enggan Jawab
H109	Dalam tempoh **12 bulan yang lepas**, selepas sesi meminum minuman yang memabukkan dalam jumlah melebihi dari biasa, berapa kerapkah pada pagi esoknya anda perlu meminum minuman yang memabukkan sebelum memulakan hari anda?<br>*Probe: Apabila anda terlebih minum engkem, berapa kerapkah anda perlu minum pada pagi hari keesokannya ?*	☐ Tidak Pernah<br>☐ Kurang dari sekali sebulan<br>☐ Sekali sebulan	☐ Sekali seminggu<br>☐ Setiap hari atau hampir setiap hari<br>☐ Tidak Tahu<br>☐ Enggan Jawab
H110	Dalam tempoh **12 bulan yang lepas**, berapa kerapkah anda rasa bersalah atau menyesal selepas minum minuman yang memabukkan?<br>*Probe: Ada rasa bersalah atau menyesal selepas minum engkem?*	☐ Tidak Pernah<br>☐ Kurang dari sekali sebulan<br>☐ Sekali sebulan	☐ Sekali seminggu<br>☐ Setiap hari atau hampir setiap hari<br>☐ Tidak Tahu<br>☐ Enggan Jawab
H111	Dalam tempoh **12 bulan yang lepas**, berapa kerapkah anda tidak dapat mengingati apakah yang telah berlaku malam sebelumnya disebabkan anda telah mengambil minuman yang memabukkan?<br>*Probe: tidak dapat ingat apa yang berlaku malam sebelumnya akibat minum engkem*	☐ Tidak Pernah<br>☐ Kurang dari sekali sebulan<br>☐ Sekali sebulan	☐ Sekali seminggu<br>☐ Setiap hari atau hampir setiap hari<br>☐ Tidak Tahu<br>☐ Enggan Jawab
H112	Pernahkah anda atau orang lain tercedera disebabkan anda meminum minuman yang memabukkan?	☐ Tidak<br>☐ Ya, tetapi bukan dalam tempoh setahun yang lepas<br>☐ Ya, dalam tempoh setahun yang lepas<br>☐ Tidak Tahu<br>☐ Enggan Jawab	

H113	Pernahkah saudara atau kawan atau doktor atau anggota kesihatan mengambil berat atau mencadangkan supaya anda mengurangkan pengambilan minuman yang memabukkan?<br><br>*Probe: Ada sesiapa yang cadangkan supaya anda kurangkan minum engkem contohnya keluarga, kawan atau doktor?*	☐ Tidak<br>☐ Ya, tetapi bukan dalam tempoh setahun yang lepas<br>☐ Ya, dalam tempoh setahun yang lepas<br>☐ Tidak Tahu<br>☐ Enggan Jawab
H114	Adakah anda masih mengambil minuman yang memabukkan dalam masa **1 bulan yang lalu**?	☐ Ya<br>☐ Tidak<br>☐ Tidak Tahu<br>☐ Enggan Jawab
**Tamat, ke Modul seterusnya**		

MODUL I: MEROKOK

**KRITERIA KELAYAKAN: AHLI ISIRUMAH BERUMUR 13 TAHUN DAN KE ATAS**		
I101	Pada ketika ini, adakah anda menghisap rokok (rokok yang dikilang, rokok gulung sendiri, kretek, curut, shisha, bidis atau paip tembakau) setiap hari, kurang daripada setiap hari, atau tidak sama sekali?<br><br>**[Sila tunjuk kad imbasan]**	**1.** Setiap hari<br>2. Kurang daripada setiap hari } **Sila ke I103**<br>3. Tidak sama sekali<br>(-7) TT (-9) EJ<br>**…sila ke I104 jika TT / EJ**
I102	Pada masa lalu, pernahkah anda menghisap rokok setiap hari, kurang daripada setiap hari, atau tidak sama sekali?	1. Setiap hari<br>2. Kurang daripada setiap hari<br>3. Tidak sama sekali } **Sila ke I104**<br>(-7) TT (-9) EJ<br>**…sila ke I104 jika TT / EJ**
I103	Pada ketika ini, antara produk tembakau berikut yang manakah anda hisap?<br><br>**[Sila tunjuk kad imbasan]**	a. Rokok yang dikilang<br>1. Ya<br>2. Tidak<br>(-7) TT (-9) EJ<br><br>b. Rokok gulung sendiri<br>1. Ya<br>2. Tidak<br>(-7) TT (-9) EJ<br><br>c. Kretek<br>1. Ya<br>2. Tidak<br>(-7) TT (-9) EJ
I104	Pada ketika ini, antara produk tembakau tanpa asap berikut yang manakah anda gunakan?<br><br>**[Sila tunjuk kad imbasan]**	
I104a	Rokok elektronik (*Vape*)?<br>1. Ya<br>2. Tidak<br>(-7) TT (-9) EJ	**I104b** Mengunyah tembakau (sentil atau songel)?<br>1. Ya<br>2. Tidak<br>(-7) TT (-9) EJ
I104c	Menghidu tembakau?<br>1. Ya<br>2. Tidak<br>(-7) TT (-9) EJ	
**Tamat, ke Modul seterusnya**		

MODUL J: TUBERKULOSIS

**KRITERIA KELAYAKAN: AHLI ISIRUMAH BERUMUR 15 TAHUN DAN KE ATAS**		
**TUBERKULOSIS (TB)**		
Sekarang, saya ingin bertanyakan tentang penyakit tuberkulosis (TB) dan tanda atau gejala penyakit tersebut,		
J101	Adakah anda pernah diberitahu anda menghidap tuberkulosis?	1. Ya<br>2. Tidak<br>(-7) TT (-9) EJ
J102	Adakah anda sedang mendapat rawatan tuberkulosis?<br><br>**[Sila rujuk buku Directly Observed Treatment Short-course (DOTS)**	1. Ya<br>2. Tidak<br>(-7) TT (-9) EJ
Dalam tempoh satu (1) bulan lepas, adakah anda menghadapi gejala seperti berikut?		
J103	Batuk lebih dari dua (2) minggu	1. Ya<br>2. Tidak<br>(-7) TT (-9) EJ
J104	Batuk berkahak lebih dari dua (2) minggu	1. Ya<br>2. Tidak<br>(-7) TT (-9) EJ
J105	Batuk berdarah	1. Ya<br>2. Tidak<br>(-7) TT (-9) EJ
J106	Demam lebih dari dua (2) minggu	1. Ya<br>2. Tidak<br>(-7) TT (-9) EJ
J107	Turun berat badan yang tidak disangka	1. Ya<br>2. Tidak<br>(-7) TT (-9) EJ
J108	Berpeluh pada waktu malam lebih dari dua (2) minggu	1. Ya<br>2. Tidak<br>(-7) TT (-9) EJ
J109	Jika anda mempunyai salah satu tanda gejala di atas, adakah anda/ anak anda mendapatkan rawatan?	1. Ya<br>2. Tidak<br>(-7) TT (-9) EJ
**Tamat, ke Modul seterusnya**		

MODUL K: KESIHATAN WANITA & AMALAN PERANCANG KELUARGA

**KRITERIA KELAYAKAN: WANITA YANG BERUMUR 15 - 49 TAHUN**		
K101	Adakah anda pernah atau sedang mengandung?	1. Ya<br>2. Tidak …**sila ke K106**<br>(-7) TT (-9) EJ
K102	Berapakah umur anda semasa mengandung kali pertama?	Umur: [ ][ ] Tahun<br>(-7) TT (-9) EJ
K103	Berapa orangkah anak kandung anda yang lahir hidup?	Bilangan: [ ][ ] Orang<br>(-7) TT (-9) EJ
K104	Di manakah anda melahirkan **anak terakhir**?	1. Klinik/ Hospital/ Pusat Transit/ Balai Rawatan …**sila ke K200**<br>2. Rumah<br>3. Tempat lain<br>4. Tidak pernah melahirkan anak …**sila ke K200**<br>(-7) TT (-9) EJ<br>**…sila ke K200 jika TT/EJ**
K105	Jika anda melahirkan anak di rumah/tempat lain, siapakah yang membantu anda menyambut kelahiran anak anda?<br>*Probe: Siapa yang sambut bayi masa beranak?*	1. Kakitangan kesihatan KKM<br>2. Bidan kampung<br>3. Pasangan<br>4. Ibu bapa<br>5. Lain-lain<br>(1–5) **Sila ke K200**<br>(-7) TT (-9) EJ<br>**…sila ke K200 jika TT/EJ**
K106	Jika tidak, adakah anda merancang untuk mengandung pada masa terdekat?	1. Ya<br>2. Tidak<br>(-7) TT (-9) EJ
K200	Pernahkah puan atau pasangan menggunakan apa-apa kaedah perancang keluarga (cara elak mengandung) bagi mengelak kehamilan?<br>*Probe: Pernah guna apa-apa cara nak elak mengandung?*	1. Ya<br>2. Tidak …**sila ke K205**<br>(-7) TT (-9) EJ
K201	Pada umur berapakah puan pertama kali menggunakan kaedah perancang keluarga (cara elak mengandung)?	Umur: [ ][ ] Tahun<br>(-7) TT (-9) EJ
K202	Berapa orangkah anak puan ketika kali pertama puan menggunakan kaedah perancang keluarga (cara elak mengandung)?<br>*Probe: Pertama kali guna cara elak mengandung masa ada anak berapa orang?*	Bilangan: [ ][ ] Orang ("0" jika tiada anak)<br>(-7) TT (-9) EJ
K203	Adakah puan/pasangan masih menggunakan apa-apa kaedah perancang keluarga (cara elak mengandung)?<br>*Probe: Sekarang ada guna apa-apa cara nak elak mengandung?*	1. Ya<br>2. Tidak, sebab sedang mengandung …**sila ke K206**<br>3. Tidak, sebab telah berhenti guna …**sila ke K205**<br>(-7) TT (-9) EJ

K204	Jika Ya, apakah kaedah perancang keluarga (cara elak mengandung) terkini yang anda pilih?<br><br>*Probe: Cara apa yang puan guna?*<br><br>**[Pilih SATU jawapan sahaja]**<br><br>**[Sila tunjuk kad imbasan]**	1. Pil perancang<br>2. Suntikan<br>3. Implan<br>4. Kondom<br>5. Alat dalam Rahim<br>6. Pembedahan tubal ligasi (ikat untuk perempuan<br>7. Vasektomi (ikat untuk lelaki)<br>8. Cara waktu selamat<br>9. Azal (pancut luar)<br>10. LAM – menyusu badan<br>11. Tradisional (Jamu, akar kayu,<br>12. urut, ubat-ubatan sendiri)<br>13. Lain-lain<br>} **Sila ke K206**<br><br>(-7) TT (-9) EJ
K205	Mengapa puan atau pasangan **TIDAK** menggunakan sebarang kaedah perancang keluarga (cara elak mengandung)?<br><br>*Probe: Kenapa tak ada guna apa-apa cara nak elak mengandung?*<br><br>**[Pilih SATU jawapan sahaja]**	1. Ingin hamil<br>2. Kesan sampingan<br>3. Nasihat pengamal perubatan<br>4. Kos yang mahal<br>5. Masalah mendapatkan bekalan<br>6. Telah putus haid<br>7. Tidak selesa<br>8. Suami tidak benarkan<br>9. Kepercayaan (agama)<br>10. Lain-lain<br><br>(-7) TT (-9) EJ
K206	Adakah puan sendiri mahu menggunakan kaedah perancang keluarga (cara elak mengandung)?<br><br>*Probe: Puan sendiri nak guna tak cara elak mengandung?*	1. Ya<br>2. Tidak<br><br>(-7) TT (-9) EJ
K207	Siapakah yang membuat keputusan dalam menggunakan kaedah perancang keluarga (cara elak mengandung) sekarang?<br><br>**[Pilih SATU jawapan sahaja]**	1. Sendiri<br>2. Pasangan/Suami<br>3. Responden dan pasangan bersama<br>4. Ahli keluarga<br>5. Jururawat/doktor<br>6. Lain-lain<br><br>(-7) TT (-9) EJ
K208	Adakah puan berhasrat menggunakan sebarang kaedah perancang keluarga (cara elak mengandung) di masa hadapan?<br><br>*Probe: Puan ada nak guna apa-apa cara elak mengandung tak nanti?*	1. Ya<br>2. Tidak<br><br>(-7) TT (-9) EJ
**Tamat, ke Modul seterusnya**		

MODUL L: KENCING MANIS

KRITERIA KELAYAKAN: AHLI ISIRUMAH BERUMUR 18 TAHUN DAN KE ATAS

Sekarang saya ingin bertanya mengenai kesihatan anda, terutamanya berkenaan kencing manis.

L101	Dalam tempoh 12 bulan yang lepas, pernahkah anda menjalani pemeriksaan paras gula dalam darah?		1. Ya<br>2. Tidak<br>(-7) TT (-9) EJ
L102	Pernahkah anda diberitahu oleh doktor ataupun Penolong Pegawai Perubatan (PPP) bahawa anda menghidap penyakit kencing manis atau diabetes?		1. Ya<br>2. Tidak …**tamat, ke Modul seterusnya**<br>(-7) TT (-9) EJ<br>**…sila ke Modul seterusnya jika TT/EJ**
L103	Jika Ya, bilakah anda diberitahu oleh doktor / PPP bahawa anda menghidap penyakit kencing manis atau diabetes?		1. <1 tahun …**sila ke L104**<br>2. ≥1 tahun<br>(-7) TT (-9) EJ<br>**…sila ke L104 jika TT/EJ**
L103a	Sila nyatakan: [ ] Tahun		(-7) TT (-9) EJ
L104	Apakah jenis rawatan atau nasihat yang anda terima daripada doktor (atau anggota kesihatan lain) untuk mengawal penyakit kencing manis?		
L104a	Insulin<br>**[Sila tunjuk kad imbasan]**<br>1. Ya<br>2. Tidak<br>(-7) TT (-9) EJ	L104b	Ubat-ubatan dalam masa 2 minggu lepas<br>1. Ya<br>2. Tidak<br>(-7) TT (-9) EJ
L104c	Nasihat diet khusus untuk penyakit kencing manis<br>1. Ya<br>2. Tidak<br>(-7) TT (-9) EJ	L104d	Nasihat untuk kurangkan berat badan<br>1. Ya<br>2. Tidak<br>(-7) TT (-9) EJ
L104e	Nasihat untuk mula bersenam atau lebihkan senaman<br>1. Ya<br>2. Tidak<br>(-7) TT (-9) EJ		
L105	Adakah anda mengambil apa-apa rawatan herbal / tradisional untuk penyakit kencing manis atau diabetes anda?<br>1. Ya<br>2. Tidak<br>(-7) TT (-9) EJ		
L106	Dimanakah anda selalunya mendapat rawatan untuk penyakit kencing manis?<br>1. Klinik kerajaan/ Klinik bergerak<br>2. Klinik swasta<br>3. Hospital kerajaan<br>4. Hospital swasta<br>5. Farmasi (rawatan sendiri)<br>6. Pengamal rawatan tradisional, herba atau komplementari<br>7. Saya tidak mendapatkan sebarang rawatan<br>(-7) TT (-9) EJ		

Tamat, ke Modul seterusnya

MODUL M: TEKANAN DARAH TINGGI

**KRITERIA KELAYAKAN: AHLI ISIRUMAH BERUMUR 18 TAHUN DAN KE ATAS**			
Sekarang saya ingin bertanya mengenai kesihatan anda, terutamanya berkenaan tekanan darah tinggi.			
M101	Dalam tempoh 12 bulan yang lepas, pernahkah anda menjalani pemeriksaan tekanan darah?		1. Ya<br>2. Tidak<br>(-7) TT (-9) EJ
M102	Pernahkah anda diberitahu oleh doktor ataupun Penolong Pegawai Perubatan (PPP) bahawa tekanan darah anda adalah tinggi atau menghidap tekanan darah tinggi?		1. Ya<br>2. Tidak …**tamat, ke Modul seterusnya**<br>(-7) TT (-9) EJ<br>**…sila ke Modul seterusnya jika TT / EJ**
M103	Jika Ya, bilakah anda diberitahu oleh doktor / PPP bahawa tekanan darah anda adalah tinggi atau menghidap tekanan darah tinggi?		1. <1 tahun …**sila ke M104**<br>2. ≥1 tahun<br>(-7) TT (-9) EJ<br>**…sila ke M104 jika TT / EJ**
M103a	Sila nyatakan: [ ] Tahun<br>(-7) TT (-9) EJ		
M104	Apakah jenis rawatan atau nasihat yang anda terima daripada doktor (atau anggota kesihatan lain) untuk mengawal tekanan darah tinggi?		
M104a	Ubat-ubatan sejak 2 minggu lepas<br>1. Ya<br>2. Tidak<br>(-7) TT (-9) EJ	M104b	Nasihat untuk kurangkan garam dalam makanan.<br>1. Ya<br>2. Tidak<br>(-7) TT (-9) EJ
M104c	Nasihat untuk kurangkan berat badan.<br>1. Ya<br>2. Tidak<br>(-7) TT (-9) EJ	M104d	Nasihat untuk mula bersenam atau lebihkan senaman.<br>1. Ya<br>2. Tidak<br>(-7) TT (-9) EJ
M105	Adakah anda mengambil apa-apa rawatan herbal / tradisional untuk penyakit darah tinggi anda?<br>1. Ya<br>2. Tidak<br>(-7) TT (-9) EJ		
M106	Dimanakah anda selalunya mendapat rawatan untuk penyakit darah tinggi?<br>1. Klinik kerajaan/ Klinik bergerak<br>2. Klinik swasta<br>3. Hospital kerajaan<br>4. Hospital swasta<br>5. Farmasi (rawatan sendiri)<br>6. Pengamal rawatan tradisional, herba atau komplementari<br>7. Saya tidak mendapatkan sebarang rawatan<br>(-7) TT (-9) EJ		
**Tamat, ke Modul seterusnya**			

MODUL N: PARAS KOLESTEROL TINGGI

KRITERIA KELAYAKAN: AHLI ISIRUMAH BERUMUR 18 TAHUN DAN KE ATAS

Sekarang saya ingin bertanya mengenai kesihatan anda, terutamanya berkenaan paras kolesterol (lemak) yang tinggi.

N101	Dalam tempoh 12 bulan yang lepas, pernahkah anda menjalani pemeriksaan kolesterol (lemak) dalam darah?	1. Ya<br>2. Tidak<br>(-7) TT (-9) EJ	
N102	Pernahkah anda diberitahu oleh doktor ataupun Penolong Pegawai Perubatan (PPP) bahawa paras kolesterol (lemak) darah anda adalah tinggi?	1. Ya<br>2. Tidak …**tamat, ke Modul seterusnya**<br>(-7) TT (-9) EJ<br>**… tamat, sila ke Modul seterusnya jika TT / EJ**	
N103	Jika Ya, bilakah anda diberitahu oleh doktor / PPP bahawa paras kolesterol (lemak) darah anda adalah tinggi?	1. <1 tahun …**sila ke N104**<br>2. ≥1 tahun<br>(-7) TT (-9) EJ<br>**… sila ke N104 jika TT / EJ**	
N103a	Sila nyatakan: [ ] Tahun<br>(-7) TT (-9) EJ		
N104	Apakah jenis rawatan atau nasihat yang anda terima daripada doktor (atau anggota kesihatan lain) untuk mengawal paras kolesterol (lemak) tinggi?		
N104a	Ubat-ubatan sejak 2 minggu lepas.<br>1. Ya<br>2. Tidak<br>(-7) TT (-9) EJ	N104b	Nasihat diet khusus seperti rendah lemak atau rendah kolesterol.<br>1. Ya<br>2. Tidak<br>(-7) TT (-9) EJ
N104c	Nasihat untuk kurangkan berat badan.<br>1. Ya<br>2. Tidak<br>(-7) TT (-9) EJ	N104d	Nasihat untuk mula bersenam atau lebihkan senaman.<br>1. Ya<br>2. Tidak<br>(-7) TT (-9) EJ
N105	Adakah anda mengambil apa-apa rawatan herbal / tradisional untuk paras kolesterol (lemak) tinggi anda?<br>1. Ya<br>2. Tidak<br>(-7) TT (-9) EJ		
N106	Dimanakah anda selalunya mendapat rawatan untuk paras kolesterol (lemak) tinggi anda?<br>1. Klinik kerajaan/ Klinik bergerak<br>2. Klinik swasta<br>3. Hospital kerajaan<br>4. Hospital swasta<br>5. Farmasi (rawatan sendiri)<br>6. Pengamal rawatan tradisional, herba atau komplementari<br>7. Saya tidak mendapatkan sebarang rawatan<br>(-7) TT (-9) EJ		

Tamat, ke Modul seterusnya

MODUL R: REKOD IMUNISASI KANAK-KANAK

KRITERIA KELAYAKAN: KANAK-KANAK UMUR 12 – 59 BULAN

UNTUK KANAK-KANAK UMUR 12 – 59 BULAN, REKOD IMUNISASI PERLU DICATATKAN OLEH JURURAWAT

***[Rujuk Buku Rekod Kesihatan Bayi Dan Kanak-kanak]**

R100	Buku Rekod Kesihatan Bayi Dan Kanak-kanak	1. Ada<br>2. Tiada …**tamat, ke Modul seterusnya**<br>(-8) Tidak berkaitan
R101	BCG	1. Ya<br>(sila nyatakan tarikh)<br>☐☐ ☐☐ ☐☐☐☐<br>2. Tidak<br>(-8) Tidak berkaitan
R102	Hep B selepas lahir (HEP 1)	1. Ya<br>(sila nyatakan tarikh)<br>☐☐ ☐☐ ☐☐☐☐<br>2. Tidak<br>(-8) Tidak berkaitan
R103	Hep B Dos 2 (HEP 2)	1. Ya<br>(sila nyatakan tarikh)<br>☐☐ ☐☐ ☐☐☐☐<br>2. Tidak<br>(-8) Tidak berkaitan
R104	DPT-IPV/ Hib Dos 1 (DPT 1)	1. Ya<br>(sila nyatakan tarikh)<br>☐☐ ☐☐ ☐☐☐☐<br>2. Tidak<br>(-8) Tidak berkaitan
R105	DPT-IPV/ Hib Dos 2 (DPT 2)	1. Ya<br>(sila nyatakan tarikh)<br>☐☐ ☐☐ ☐☐☐☐<br>2. Tidak<br>(-8) Tidak berkaitan
R106	DPT-IPV/ Hib Dos 3 (DPT 3)	1. Ya<br>(sila nyatakan tarikh)<br>☐☐ ☐☐ ☐☐☐☐<br>2. Tidak<br>(-8) Tidak berkaitan
R107	Hep B Dos 3 (HEP 3)	1. Ya<br>(sila nyatakan tarikh)<br>☐☐ ☐☐ ☐☐☐☐<br>2. Tidak<br>(-8) Tidak berkaitan
R108	MMR	1. Ya<br>(sila nyatakan tarikh)<br>☐☐ ☐☐ ☐☐☐☐<br>2. Tidak<br>(-8) Tidak berkaitan
R109	DPT-IPV/ Hib Dos Tambahan (Booster)	1. Ya<br>(sila nyatakan tarikh)<br>☐☐ ☐☐ ☐☐☐☐<br>2. Tidak<br>(-8) Tidak berkaitan

Tamat, ke Modul seterusnya

MODUL P1: PENGUKURAN ANTROPOMETRI

KRITERIA KELAYAKAN: SETIAP AHLI ISIRUMAH

ARAHAN:

UNTUK KANAK-KANAK UMUR BAWAH 5 TAHUN (0 – 59 BULAN), BERAT, PANJANG DAN LILITAN KEPALA SEMASA LAHIR PERLU DICATATKAN OLEH JURURAWAT

***[Rujuk Buku Rekod Kesihatan Bayi Dan Kanak-kanak]**

P100	Buku Rekod Kesihatan Bayi Dan Kanak-kanak	1. Ada<br>2. Tiada …**tamat, sila ke P104**<br>(-8) Tidak berkaitan
P101	Berat semasa lahir:	☐☐☐☐ gm<br>(-8) Tidak berkaitan
P102	Panjang semasa lahir:	☐☐ . ☐ cm<br>(-8) Tidak berkaitan
P103	Lilitan kepala semasa lahir:	☐☐ . ☐ cm<br>(-8) Tidak berkaitan

Kriteria pengecualian:

Adakah responden mempunyai "kriteria pengecualian" seperti di bawah?

P104	Wanita mengandung	1. Ya<br>2. Tidak
P105	Wanita yang baru melahirkan anak (<60 hari dari tarikh lawatan)	1. Ya<br>2. Tidak
P106	Terlantar - akibat sakit teruk/berpanjangan, kecederaan / kemalangan	1. Ya<br>2. Tidak
P107	Mengalami ketidakupayaan fizikal yang menghadkan kebolehan untuk berdiri dengan tegak termasuk yang memakai kerusi roda	1. Ya<br>2. Tidak
P108	Cacat anggota badan seperti tiada tangan atau tiada kaki, spondylosis (bengkok tulang belakang) kecuali buta, bisu dan pekak	1. Ya<br>2. Tidak

Jika 'Ya' kepada salah satu pilihan di atas, tamat Modul P1 dan terus ke Modul P2 (jika wanita berumur 15-49 tahun) atau P3 (jika responden berumur 18 tahun dan ke atas).

P109	**Tarikh Pengukuran Antropometri:** Hari / Bulan / Tahun			
	**Berat Badan:**			
P110	Ukuran 1	. kg	(-8) Tidak berkaitan	
P111	Ukuran 2	. kg	(-9) Enggan diukur	
	**Panjang / Tinggi:**			
P112	Ukuran 1	. cm	(-8) Tidak berkaitan	
P113	Ukuran 2	. cm	(-9) Enggan diukur	
	***Panjang: Kanak-kanak berumur bawah 2 tahun (diukur secara berbaring)**			
	**Ukur lilit pinggang:**			
	**KRITERIA KELAYAKAN: UNTUK RESPONDEN BERUMUR 18 TAHUN DAN KE ATAS**			
P114	Ukuran 1	. cm	(-8) Tidak berkaitan	
P115	Ukuran 2	. cm	(-9) Enggan diukur	
	**Panjang separa depa lengan:**			
	**KRITERIA KELAYAKAN: UNTUK RESPONDEN YANG TIDAK BOLEH BERDIRI TEGAK SAHAJA**			
P116	Ukuran 1	. cm	(-8) Tidak berkaitan	
P117	Ukuran 2	. cm	(-9) Enggan diukur	
	**Ukur lilit betis:**			
	**KRITERIA KELAYAKAN: UNTUK RESPONDEN BERUMUR 60 TAHUN DAN KE ATAS**			
P118	Ukuran 1	. cm	(-8) Tidak berkaitan	
P119	Ukuran 2	. cm	(-9) Enggan diukur	
	**Ukur lilit kepala:**			
	**KRITERIA KELAYAKAN: UNTUK RESPONDEN BERUMUR 5 TAHUN DAN KE BAWAH**			
P120	Ukuran 1	. cm	(-8) Tidak berkaitan	
P121	Ukuran 2	. cm	(-9) Enggan diukur	
	**Ukur lilit pergelangan lengan atas:**			
	**KRITERIA KELAYAKAN: UNTUK RESPONDEN BERUMUR 5 TAHUN DAN KE BAWAH**			
P122	Ukuran 1	. cm	(-8) Tidak berkaitan	
P123	Ukuran 2	. cm	(-9) Enggan diukur	

MODUL P2: ANEMIA: PEMERIKSAAN HEMOGLOBIN

Keseluruhan Modul ini perlu dijalankan dan diisi oleh Jururawat.

KRITERIA KELAYAKAN RESPONDEN: WANITA BERUMUR 15 – 49 TAHUN

P201	Bacaan Hemoglobin	☐☐ . ☐ g/dl<br>(-8) Tidak berkaitan<br>(-9) Enggan diperiksa

MODUL P3: PEMERIKSAAN TEKANAN DARAH

Keseluruhan Modul ini perlu dijalankan dan diisi oleh Jururawat.

KRITERIA KELAYAKAN RESPONDEN: BERUMUR 18 TAHUN DAN KE ATAS

Bacaan tekanan darah

P301	Sistolik	1	Bacaan pertama	☐☐☐
P302	Diastolik	1	Bacaan pertama	☐☐☐
P303	Sistolik	2	Bacaan kedua	☐☐☐
P304	Diastolik	2	Bacaan kedua	☐☐☐
P305	Sistolik	3	Bacaan ketiga	☐☐☐
P306	Sistolik	3	Bacaan ketiga	☐☐☐

(-8) Tidak berkaitan
(-9) Enggan diperiksa

MODUL P4: PEMERIKSAAN GLUKOSA DAN KOLESTEROL DARAH

Keseluruhan Modul ini perlu dijalankan dan diisi oleh Jururawat.

KRITERIA KELAYAKAN RESPONDEN: BERUMUR 18 TAHUN DAN KE ATAS

P401	Dalam tempoh 8 jam yang lepas, adakah anda makan dan minum selain dari air kosong?	1. Ya<br>2. Tidak<br>(-7) TT (-9) EJ
P402	Bacaan paras glukosa kapilari	☐☐☐ . ☐ mmol/L<br>(-9) Enggan diperiksa
P403	Bacaan paras kolesterol	☐☐☐ . ☐ mmol/L<br>(-9) Enggan diperiksa

MODUL P5: PEMERIKSAAN PENDEDAHAN KEPADA LOGAM BERAT		
**Pengambilan sampel kuku dan rambut responden perlu dijalankan oleh Jururawat.**		
**KRITERIA KELAYAKAN: SETIAP AHLI ISIRUMAH (UNTUK KAMPUNG ORANG ASLI YANG TERPILIH SAHAJA)**		
P501	**UNTUK DIISI OLEH PENEMURAMAH:**<br>Adakah kampung yang dilawati terpilih untuk pemeriksaan pendedahan kepada logam berat?	1. Ya<br>2. Tidak
P502	Pengambilan sampel kuku	1. Setuju<br>2. Tidak setuju
P503	Adakah pengambilan sampel kuku berjaya?	1. Berjaya<br>2. Tidak berjaya
P504	Pengambilan sampel rambut	1. Setuju<br>2. Tidak setuju
P505	Adakah pengambilan sampel rambut berjaya?	1. Berjaya<br>2. Tidak Berjaya

~ TAMAT SOAL SELIDIK ~

APPENDIX 8: QUESTIONNAIRE (HOUSEHOLD)

INSTITUTE FOR PUBLIC HEALTH @NIH

JABATAN KEMAJUAN ORANG ASLI

TINJAUAN KESIHATAN ORANG ASLI

BORANG SOAL SELIDIK
ISIRUMAH

Bil.	Tajuk Modul	Muka Surat	Catatan
A	Jadual Isirumah		
A1	Isirumah		
AB	Ketidakjaminan Makanan		

INSTITUT KESIHATAN UMUM
KEMENTERIAN KESIHATAN MALAYSIA

TINJAUAN KESIHATAN ORANG ASLI

INSTITUT KESIHATAN UMUM

KEMENTERIAN KESIHATAN MALAYSIA

BORANG SOAL SELIDIK (ISIRUMAH)

(UNTUK DIISI OLEH PENEMURAMAH)						
ID	Negeri (2 Digit)	Daerah (2 Digit)	Kawasan (1 Digit)	Kampung (2 Digit)	TK (3 Digit)	Isirumah (IR) (2 Digit)

TARIKH TEMURAMAH	Haribulan	Bulan	Tahun

STATUS TEMPAT KEDIAMAN

☐ Berjaya ☐ Gagal

Jika tempat kediaman gagal, nyatakan sebab gagal

- ☐ Tempat Kediaman Enggan
- ☐ Tempat Kediaman Berkunci
- ☐ Tempat Kediaman Kosong
- ☐ Tempat Kediaman Bukan Orang Asli
- ☐ Lain-lain, nyatakan:……………………………………

KOORDINAT LOKASI GEOGRAFI	i. Latitude	☐☐☐ . ☐☐☐☐☐☐
	ii. Longitude	☐☐☐ . ☐☐☐☐☐☐

MODUL A1: ISIRUMAH

**Soalan untuk diisi oleh penemuramah: Pilih SATU jawapan sahaja.**		
**Saya akan bertanyakan beberapa soalan mengenai tempat kediaman (rumah) anda.**		
A102	Tinggal secara:	1. Tetap …**sila ke A104**<br>2. Ada rumah tetap tetapi mempunyai kediaman lain yang tinggal lebih daripada 2 minggu ("semi-nomadik")<br>(-7) TT (-9) EJ
A103	Dalam **tempoh 6 bulan yang lepas**, jika mempunyai kediaman lain yang tinggal lebih daripada 2 minggu, adalah disebabkan…<br>**[Pilih SATU jawapan utama sahaja, iaitu untuk tempoh tinggal yang paling lama]**	1. Melawat keluarga/ kenduri<br>2. Perayaan kaum<br>3. Mencari hasil hutan/buah/pendapatan<br>4. Buka kebun<br>5. Kepercayaan adat (cth: asingkan diri untuk berubat)<br>6. Berlaku kematian/ bencana<br>7. Ada penyakit di kalangan ahli keluarga<br>8. Lain-lain<br>(-7) TT (-9) EJ
A104	Jenis rumah:<br>**[Pemerhatian oleh penemuramah]**	1. Rumah sebuah (rumah Projek Pembangunan Rakyat; PPR)<br>2. Rumah berkembar<br>3. Rumah teres setingkat<br>4. Rumah teres dua tingkat atau lebih<br>5. Pangsapuri<br>6. Rumah tradisional/pondok/buluh<br>7. Rumah kedai<br>8. Lain-lain<br>(-7) TT (-9) EJ
A105	Siapakah pemilik rumah ini?<br>**[Pilih SATU jawapan sahaja]**	1. Sendiri<br>2. Pasangan<br>3. Anak<br>4. Cucu<br>5. Ibu bapa<br>6. Mertua<br>7. Saudara mara<br>8. Orang lain yang bukan ahli keluarga<br>9. Majikan<br>10. Sewa<br>11. Lain-lain<br>(-7) TT (-9) EJ
**Soalan yang seterusnya adalah berkaitan bekalan air, sanitasi, cara pelupusan sampah dan air limbah/sisa di tempat kediaman (rumah) anda.**		
A106	Adakah terdapat paip air bermeter di luar rumah?<br>**[PENEMURAMAH: Sila tanya dan juga memerhatikan keadaan sebenar]**	1. Ya (Pemilik Meter)<br>2. Ya (Meter Kongsi)<br>3. Tidak …**sila ke A109**
A107	Adakah anda masih menggunakan air paip syarikat bekalan air?	1. Ya …**sila ke A109**<br>2. Tidak<br>(-7) TT (-9) EJ<br>**…sila ke A109 jika TT/EJ**
A108	Mengapakah anda tidak lagi menggunakannya?<br>**[Boleh pilih lebih dari satu jawapan]**	1. Perlu bayar/ bekalan air telah dipotong<br>2. Gangguan bekalan air<br>3. Tekanan air rendah<br>4. Terdapat sumber air lain (alternative) yang percuma<br>5. Tidak suka rasa/bau air<br>6. Lain-lain<br>(-7) TT (-9) EJ
A109	Apakah sumber air yang dibekalkan ke kawasan rumah anda?<br>**[Boleh pilih lebih dari satu jawapan]**<br>**[Sila tunjuk kad imbasan]**	1. Air paip syarikat bekalan air (air terawat)<br>2. Sistem Paip Graviti (*Gravity feed system*; GFS)<br>3. Telaga gali<br>4. Telaga tiub<br>5. Tadahan air hujan<br>6. Tadahan air semula jadi (Carok/Sungai/Tasik/Kolam)<br>(-7) TT (-9) EJ

A110	Pada kebiasaannya, apakah sumber air yang anda gunakan untuk minum/memasak?<br><br>**[Pilih SATU jawapan sahaja]**<br><br>**[Sila tunjuk kad imbasan]**	1. Air paip syarikat bekalan air (air terawat)<br>2. Sistem Paip Graviti (*Gravity feed system*; GFS)<br>3. Telaga gali<br>4. Telaga tiub<br>5. Tadahan air hujan<br>6. Tadahan air semula jadi (Carok/Sungai/Tasik/Kolam)<br>(-7) TT (-9) EJ
A111	Pada pendapat anda, adakah air di rumah anda selamat diminum?	1. Ya …**sila ke A113**<br>2. Tidak<br>(-7) TT (-9) EJ<br>**…sila ke A113 jika TT/EJ**
A112	Jika Tidak, mengapa?<br><br>**[Boleh pilih lebih dari satu jawapan]**	1. Tidak nampak bersih<br>3. Berbau (contohnya, berbau lumpur, kimia)<br>4. Rasa tak sedap<br>5. Air tidak dirawat<br>6. Lain-lain<br>(-7) TT (-9) EJ
A113	Adakah anda memasak air sebelum minum?	1. Tidak masak<br>2. Kadang-kadang<br>3. Sentiasa (Bekalan air dipastikan telah masak)<br>} **Sila ke A115** (2, 3)<br>(-7) TT (-9)EJ<br>**…sila ke A115 jika TT/EJ**
A114	Jika tidak, apakah sebab anda tidak memasak air?<br><br>**[Boleh pilih lebih dari satu jawapan]**	1. Selamat diminum tanpa perlu dimasak<br>2. Air yang dimasak tidak sedap<br>3. Malas masak air<br>4. Masalah bahan bakar<br>5. Menggunakan sistem penapis air<br>(-7) TT (-9) EJ
A115	Pada kebiasaannya, di manakah tempat anda memasak?	1. Dalam rumah<br>2. Luar rumah<br>(-7) TT (-9) EJ
A116	Apakah jenis bahan api yang digunakan?	1. Kayu/ Arang/ Sabut/ Daun kering<br>2. Gas<br>3. Elektrik<br>4. Lain-lain<br>(-7) TT (-9) EJ
A117	Apakah jenis tandas di rumah anda?<br><br>**[Sila tunjuk kad imbasan]**	1. Tandas curah<br>2. Tandas pam<br>3. Tandas lubang bertutup (*Pit latrine*)<br>5. Tandas lubang tidak bertutup<br>6. Tandas gantung (*Hanging latrine*)<br>**7.** Tiada tandas<br>(-7) TT (-9) EJ
A118	Bagaimanakah anda melupuskan sampah di rumah anda?<br><br>**[Boleh pilih lebih dari satu jawapan]**<br><br>**[Sila tunjuk kad imbasan]**	1. Bakar sendiri<br>2. Bakar di rumah bakar sampah<br>3. Lubang kambus bertutup<br>4. Lubang sampah tidak bertutup<br>5. Kutipan sampah rumah ke rumah oleh Pihak Berkuasa Tempatan (PBT)<br>6. Tong sampah komunal PBT dengan adanya tong sampah untuk simpanan sementara di kawasan rumah<br>7. Buang di luar rumah/sungai/parit<br>(-7) TT (-9) EJ
A119	Bagaimanakah sisa air buangan dari tempat penyediaan makanan (dapur) dibuang?<br><br>**[PENEMURAMAH: Sila tanya dan juga memerhatikan keadaan sebenar]**<br><br>**[Sila tunjuk kad imbasan]**	1. Bersistem<br>2. Bersistem tetapi tidak memuaskan<br>3. Tidak bersistem<br>(-7) TT (-9) EJ
A120	Adakah keluarga anda mendapat bantuan makanan setiap bulan?	1. Ya<br>2. Tidak<br>(-7) TT (-9) EJ
**Tamat, ke Modul seterusnya**		

MODUL AB: KETIDAKJAMINAN MAKANAN		
**KRITERIA KELAYAKAN: SOALAN INI PERLU DITANYA KEPADA IBU (SEKIRANYA IBU TIDAK DAPAT MENJAWAB, BAPA BOLEH JAWAB).**		
Sekarang saya akan membaca beberapa kenyataan mengenai keadaan pemakanan anda. Untuk kenyataan-kenyataan ini, sila jawab untuk dalam **tempoh 12 bulan yang lepas**, iaitu dari bulan (nama bulan semasa).		
AB01	Anda atau ahli-ahli dalam isirumah anda risau tidak mempunyai makanan yang cukup kerana kekurangan wang atau sumber-sumber lain?<br>*Probe:*<br>*1. Pernah tak tuan/puan mengalami tak cukup makanan?*<br>*2. Risau /bimbang / takut atau tak kalau x cukup makanan?*<br>*3. Contoh sumber lain: hasil hutan/ kerja/ sumbangan /bantuan*	1. Ya<br>2. Tidak<br>(-7) TT (-9) EJ
AB02	Masih memikirkan 12 BULAN yang lepas, adakah anda atau ahli-ahli dalam isirumah anda tidak dapat makan makanan yang sihat dan berkhasiat kerana kekurangan wang atau sumber-sumber lain?<br>*Probe:*<br>*1. Selalunya waktu pagi/tgh/malam, makan apa?*<br>*2. Definisi makanan sihat: dalam setiap hidangan makanan hendaklah mengandungi sumber karbohidrat/kanji, sumber protein dan sumber fiber (sayur atau buah)*<br>*3. Definisi makanan berkhasiat: tiada makanan ringan eg junk foods/keropok/kerepek*	1. Ya<br>2. Tidak<br>(-7) TT (-9) EJ
AB03	Adakah anda atau ahli-ahli dalam isirumah anda makan hanya beberapa jenis makanan sahaja kerana kekurangan wang atau sumber-sumber lain?<br>*Probe:*<br>*Jika pernah tak cukup makan, bagaimana cara atasi?*<br>*1. Hanya makan 1 @ 2jenis sahaja untuk setiap kali masa makan? – type of foods*	1. Ya<br>2. Tidak<br>(-7) TT (-9) EJ
AB04	Adakah anda atau ahli-ahli dalam isirumah anda terpaksa meninggalkan satu waktu makan kerana kekurangan wang atau sumber-sumber lain untuk mendapatkan makanan?<br>*Probe:*<br>*Jika pernah tak cukup makan, bagaimana cara atasi?*<br>*1. Kurangkan bilangan makanan yang diambil*<br>*(e.g. hanya sarapan sahaja & ini perlu mengikut rutin pengambilan makanan harian individu) – skip meals*	1. Ya<br>2. Tidak<br>(-7) TT (-9) EJ
AB05	Masih memikirkan 12 BULAN yang lepas, adakah anda atau ahli ahli dalam isirumah anda makan kurang daripada apa yang sepatutnya anda makan kerana kekurangan wang atau sumber-sumber lain?<br>*Probe:*<br>*Jika pernah tak cukup makan, bagaimana cara atasi?*<br>*1. Kurangkan porsi/saiz/sukatan makanan - portion*	1. Ya<br>2. Tidak<br>(-7) TT (-9) EJ
AB06	Adakah isirumah anda kehabisan makanan kerana kekurangan wang atau sumber-sumber lain?<br>*Probe: Pernah atau tidak di rumah tiada makanan langsung?*	1. Ya<br>2. Tidak<br>(-7) TT (-9) EJ
AB07	Adakah anda atau ahli-ahli dalam isirumah anda berasa lapar tetapi tidak makan kerana tidak mempunyai wang atau sumber-sumber lain yang cukup untuk makanan?<br>*Probe: Pernah atau tak rasa lapar dan tidak dapat makan?*	1. Ya<br>2. Tidak …**sila ke AB08**<br>(-7) TT (-9) EJ
AB07a	Sepanjang 12 bulan yang lepas, berapa kerapkah anda atau ahli-ahli dalam isirumah anda berasa lapar tetapi tidak makan kerana tidak mempunyai wang atau sumber-sumber lain yang cukup untuk makanan?<br>*Probe: Berapa kerap/berapa kali*<br>**[PENEMURAMAH: Jika responden mengatakan ini tidak berlaku dalam 12 bulan yang lepas, kembali ke AB07 dan kod sebagai “Tidak”]**	1. Hanya sekali atau dua kali<br>2. Dalam beberapa bulan tetapi bukan setiap bulan<br>3. Hampir setiap bulan<br>(-7) TT (-9) EJ
AB08	Adakah anda atau ahli-ahli dalam isirumah anda tidak makan sepanjang hari kerana kekurangan wang atau sumber-sumber lain?<br>*Probe: Pernah atau tidak anda seharian tidak dapat makan?*	1. Ya<br>2. Tidak …**tamat, ke Modul seteusnya**<br>(-7) TT (-9) EJ
AB08a	Sepanjang 12 bulan yang lepas, berapa kerapkah ianya berlaku bila anda atau ahli-ahli dalam isirumah anda tidak makan sepanjang hari kerana kekurangan wang atau sumber-sumber lain?<br>*Probe: Berapa kerap/berapa kali*<br>**[PENEMURAMAH: Jika responden mengatakan ini tidak berlaku dalam 12 bulan yang lepas, kembali ke AB08 dan kod sebagai “Tidak”]**	1. Hanya sekali atau dua kali<br>2. Dalam beberapa bulan tetapi bukan setiap bulan<br>3. Hampir setiap bulan<br>(-7) TT (-9) EJ
**Tamat, sila ke Modul Individu**		

APPENDIX 9: CODE BOOK

ISI KANDUNGAN

Bil.	Modul	Tajuk Modul	Muka Surat
1	A1	Isirumah<br>• A109: Jenis-jenis sistem bekalan air<br>• A117: Jenis-jenis tandas<br>• A118: Jenis-jenis sistem pelupusan sampah<br>• A119: Jenis-jenis pelupusan aur sisa dapur	 2<br>3<br>4<br>5
2	B	Tingkah Laku Mendapatkan (Jagaan) Kesihatan & Penggunaan Perkhidmatan Kesihatan<br>• Kod A – Masalah Kesihatan<br>• Kod B – Sebab Dimasukkan ke wad	 6<br>7
3	C	Kesihatan Mental Dewasa (GHQ-12)	8
4	F	Kesihatan Gigi dan Mulut<br>• F101 & F102: Amalan Menggosok gigi<br>• F201: Berjumpa doktor gigi/ misi gigi<br>• F301 & F302: Nasihat/ maklumat mengenai penjagaan gigi<br>• F401 & F402: Amalan mengunyah daun sirih	 9<br>9<br>9<br>9
5	H	Alkohol	10
6	I	Merokok<br>• I101 & I103: Jenis-jenis produk tembakau yang dihisap<br>• I104: Jenis-jenis produk tembakau tanpa asap	 11<br>12
7	K	Kesihatan Wanita & Amalan Perancang Keluarga<br>• K204: Jenis – jenis alat pencegah kehamilan	13
8	L	Kencing Manis<br>• L104a: Insulin	14

Modul A1: Isirumah

A109 & A110: Jenis – jenis sistem bekalan air

<table>
<tr><td>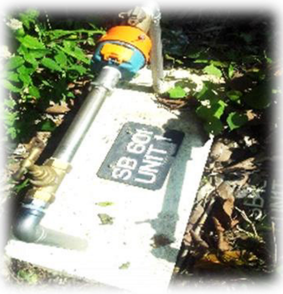</td><td>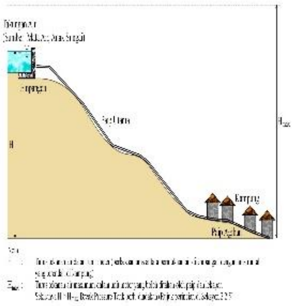</td><td>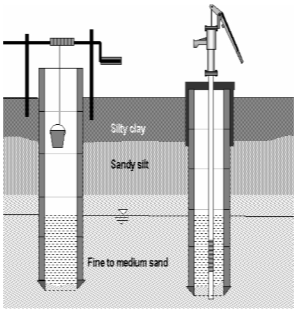
</td></tr>
<tr><td>Air paip syarikat bekalan air (air terawat)</td><td>Sistem Tadahan Graviti</td><td>Telaga terbuka</td></tr>
</table>

<table>
<tr><td>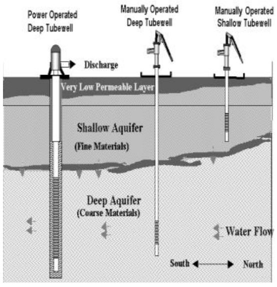
</td><td>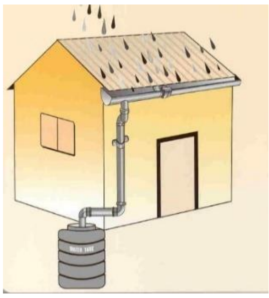</td><td>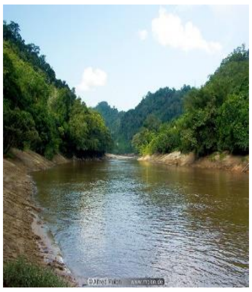</td></tr>
<tr><td>Perigi tiub</td><td>Tadahan air hujan</td><td>Tadahan air semula jadi (Carok/Sungai/Tasik/ Kolam)</td></tr>
</table>

Modul A1: Isirumah

A117: Jenis – jenis tandas

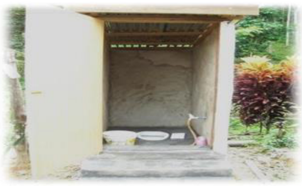

Tandas curah
(Tiada pam)

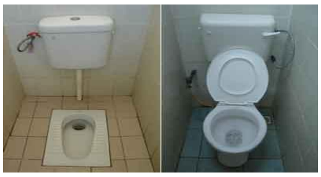

Tandas pam
(Ada pam)

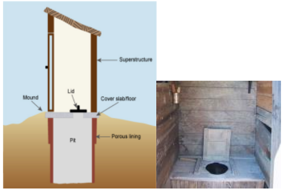

Tandas lubang bertutup
(Ada penutup)

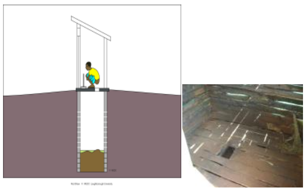

Tandas lubang tidak bertutup
(Tiada penutup)

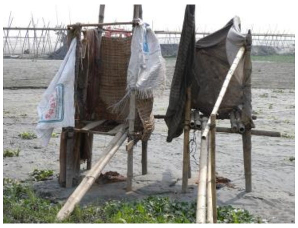

Tandas gantung
(*Hanging latrine*)

Modul A1: Isirumah

A118: Jenis – jenis sistem pelupusan sampah

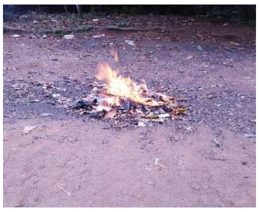	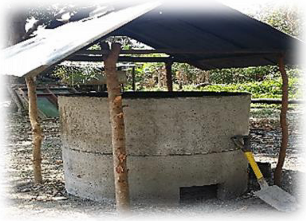	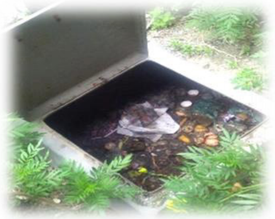
Bakar sendiri	Bakar di rumah bakar sampah	Lubang/Kambus bertutup

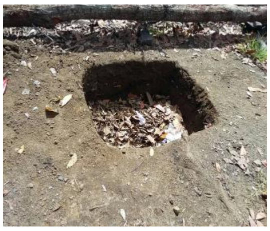	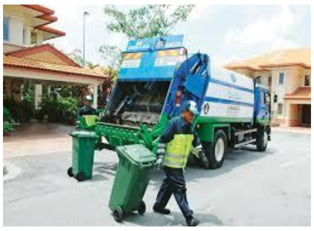	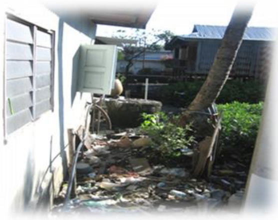
Lubang tidak bertutup	Pihak Berkuasa Tempatan (PBT)	Buang di luar rumah

Modul A1: Isirumah

A119: Jenis – jenis sistem pelupusan air sisa dapur

Bersistem	
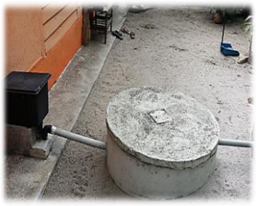	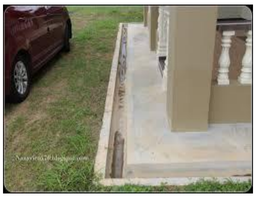
Lubang serapan (Tidak penuh)	Longkang luar rumah (Tiada sisa)

Tidak bersistem			
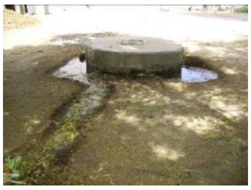	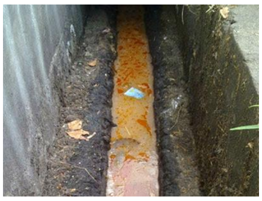	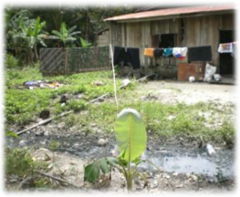	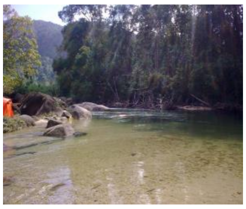
Longkang serapan melimpah	Air sisa bertakung	Salur belakang rumah	Basuh di sungai

Modul B: Tingkah Laku Mendapatkan (Jagaan) Kesihatan & Penggunaan Perkhidmatan Kesihatan

Kod A – Masalah kesihatan

Soalan B313 dan B500
(Rujukan: Buku Kod NHMS 2019, Institut Kesihatan Umum.)

Kod	Pilihan Jawapan
01	Demam/ *Fever*
02	Sakit tekak / *Sore throat*
03	Sukar untuk menelan/ *Difficulty in swallowing*
04	Selsema/Hidung tersumbat/ *Running nose/Blocked nose*
05	Batuk, berkahak atau tanpa kahak/ *Cough (with or without phlegm)*
06	Nafas berbunyi/Asma/ *Wheezing/Asthma*
07	Sakit telinga/Telinga bernanah/ *Earache/Pus from ear(s)*
08	Sakit mata/Radang mata/ *Conjunctivitis*
09	Sakit perut/ *Stomach ache*
10	Kembung perut/ *Bloated stomach*
11	Cirit birit/ *Diarrhoea*
12	Muntah/ *Vomiting*
13	Sakit ulu hati/ *Heartburn*
14	Masalah kencing/ *Urination problem*
15	Bengkak/ *Swelling*
16	Masalah kulit/Ekzema/ *Skin problem/Eczema*
17	Sakit kepala/Pening Kepala/ *Headache/Dizziness/Giddiness*
18	Alahan/ *Allergies*
19	Luka/ *Skin lesion*
20	Sakit dada/ *Chest pain*
21	Sakit sendi dan/atau tulang/ *Joint and/or bone pain*
22	Lain-lain masalah/ *Others*

Modul B: Tingkah Laku Mendapatkan (Jagaan) Kesihatan & Penggunaan Perkhidmatan Kesihatan

Kod B – Sebab dimasukkan ke wad

Soalan B401
(Rujukan: Laporan Teknikal JAKOA "Health Status of Orang Asli in Peninsular Malaysia 2003-2007" dan PER-PD206 HIMS Sistem Maklumat Rawatan Perubatan (2010, 2011, 2012), KKM.)

Kod	Pilihan Jawapan
01	Bersalin/<br>*Delivery – ICD10 O80-O84*
02	Sakit kuning bayi baru lahir/<br>*Neonatal Jaundice; all causes – ICD10 P55-P59*
03	Kusta/<br>*Leprosy – ICD10 A30*
04	Batuk berdarah/Tuberkulosis/<br>*Pulmonary Tuberculosis – ICD10 A15-A16*
05	Radang/jangkitan paru-paru, paru-paru berair/<br>*Pneumonia, Chronic lower respiratory diseases – ICD10 J12-J18, J40-J47*
06	Jangkitan saluran pernafasan atas akut/<br>*Acute upper respiratory tract infections; nasopharyngitis/ sinusitis/ pharyngitis/ tonsillitis/ laryngitis/ tracheitis/ combination – ICD10 J00-J06*
07	Sakit perut dan cirit-birit/mual/muntah/<br>*Acute gastroenteritis – ICD10 A00-A09*
08	Demam Denggi/<br>*Dengue fever – ICD10 A90-A91*
09	Sakit/serangan/gagal jantung/<br>*Ischaemic heart disease, Heart failure – ICD10 I20-I25, I50*
10	Barah/kanser/<br>*Malignant cancer; all cancers – ICD10 C00-C99*
11	Rawatan/pemeriksaan susulan (contoh: ujian darah/cairan tubuh, pemindahan darah, X-Ray, CT Scan, skop melalui mulut/anus, kemoterapi)/<br>*Follow-up treatment/ examination/ Investigation – ICD10 R00-R99*
12	Lain-lain/<br>*Others*

Modul C: GHQ-12

1	2	3	4
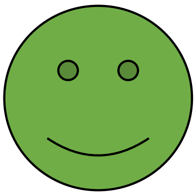	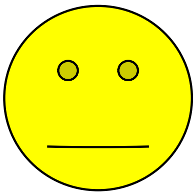	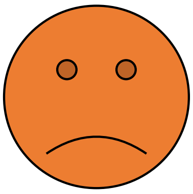	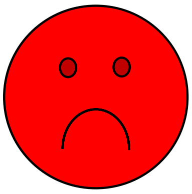
• Lebih baik dari biasa<br>• Tidak sama sekali	• Sama seperti biasa<br>• Tidak lebih dari biasa	• Kurang dari biasa<br>• Agak lebih dari biasa	• Sangat kurang dari biasa/mampu<br>• Sangat lebih dari biasa

Modul F: Kesihatan Gigi dan Mulut

F101 & F102: Amalan menggosok gigi

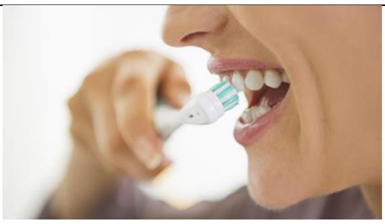

Gosok gigi dengan berus gigi

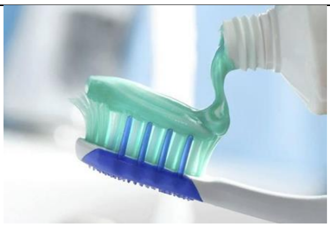

Ubat gigi

F201: Berjumpa doktor gigi/ misi gigi

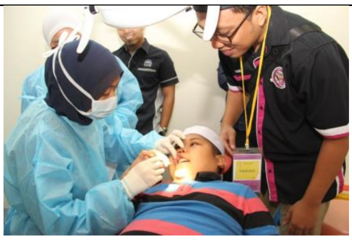

Berjumpa doktor gigi/ misi gigi

F301 & F302: Nasihat/maklumat mengenai penjagaan gigi

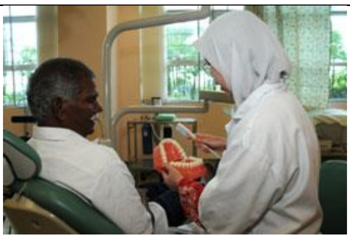

Doktor gigi/ misi gigi bagi nasihat/maklumat

F401 & F402: Amalan mengunyah daun sirih

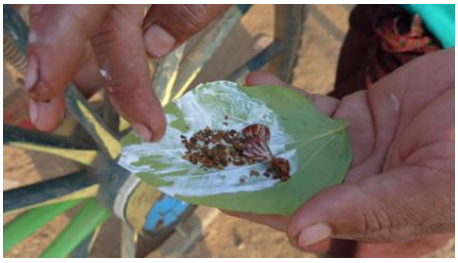

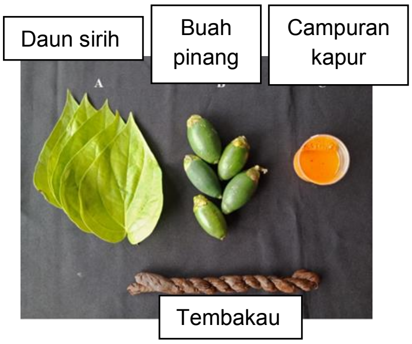

Kunyahan daun sirih bersama kapur dan buah pinang

Modul H: Alkohol/ Minuman yang memabukkan

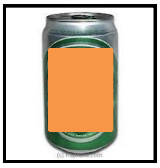

320 ml (1 tin) bir
Kandungan alkohol >2% dan <10%

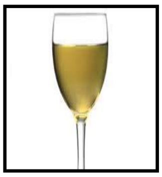

140 ml wain merah
Kandungan alkohol 7% hingga 15%

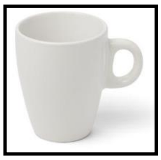

150 ml wain kelapa / bahar
Kandungan alkohol 7% hingga 15%

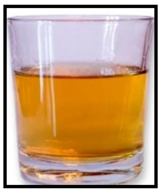

100 ml wain kelapa / lihing
Kandungan alkohol 7% hingga 15%

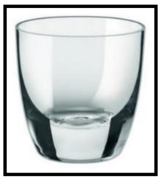

30 ml Montoku
Kandungan alkohol > 30%

30 ml Langkau / Wain beras/ Brendi Wiski
Kandungan alkohol > 30%

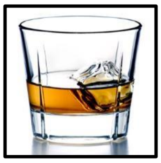

Modul I: Merokok

I101 & I103: Jenis – jenis produk tembakau yang dihisap

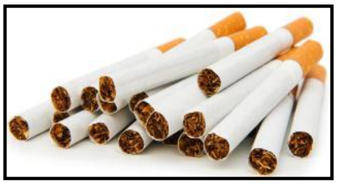

Rokok yang dikilang

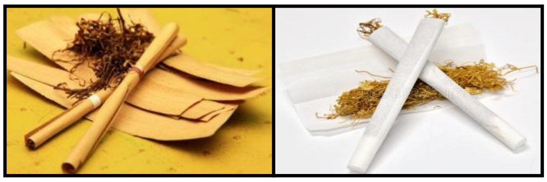

Rokok gulung sendiri

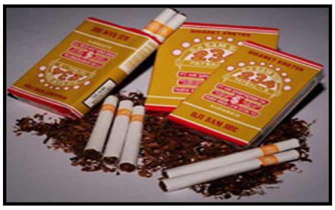

Kretek

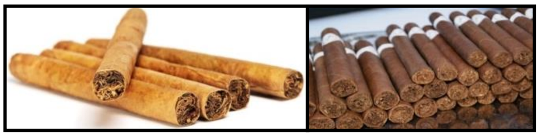

Curut

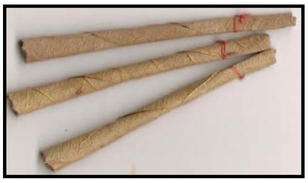

Bidis

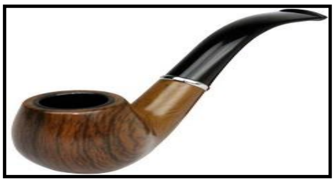

Paip tembakau

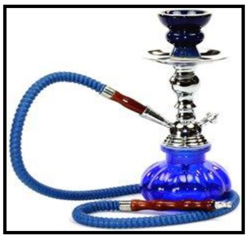

Shisha

Modul I: Merokok

I104: Jenis – jenis produk tembakau tanpa asap

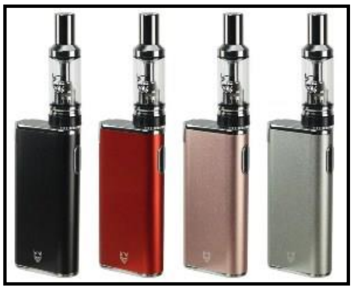

Rokok elektronik

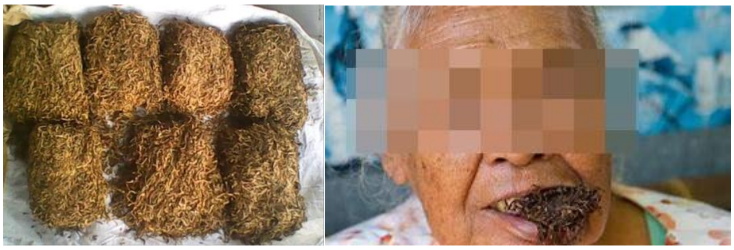

Mengunyah tembakau

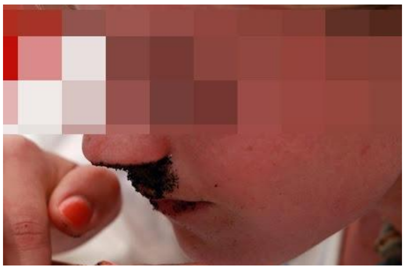

Menghidu tembakau

Modul K: Kesihatan Wanita & Amalan Perancang Keluarga

K202: Jenis – jenis alat pencegah kehamilan

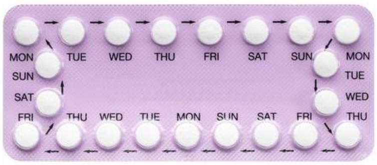	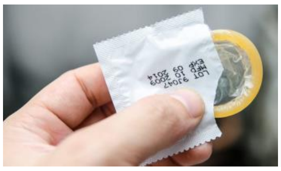 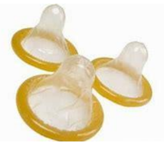
Pil perancang	Kondom
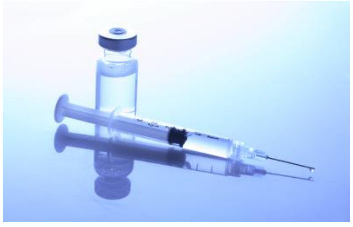	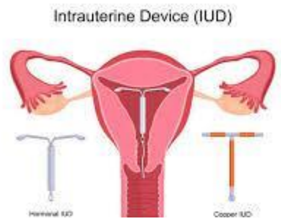
Suntikan	Alat dalam rahim/ IUCD
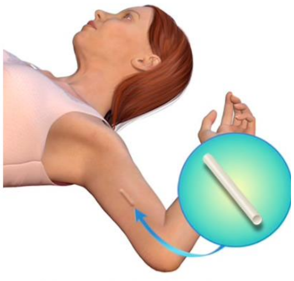	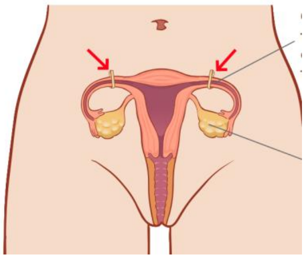
Implan	Pembedahan tubal ligase/ ikat untuk perempuan
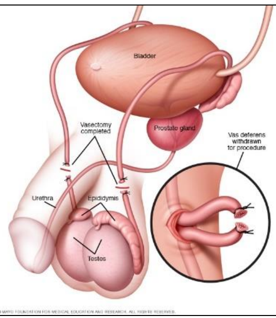	
Vasektomi/ ikat untuk lelaki	

Modul L: Kencing Manis

L104a: Insulin

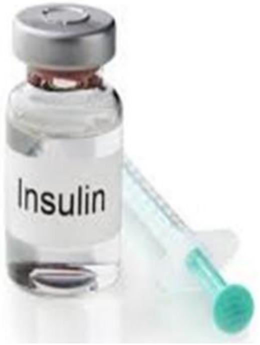

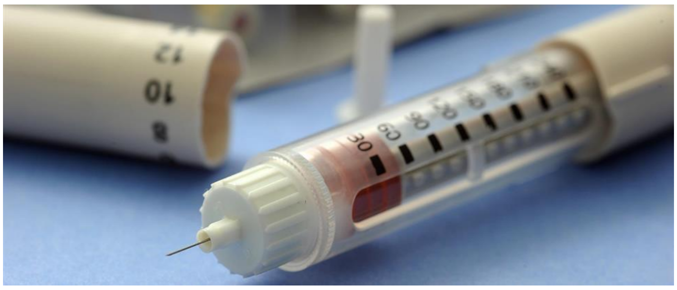

Suntikan insulin / Pen insulin

Saringan Penyakit Berjangkit

Gambar A: Yaws

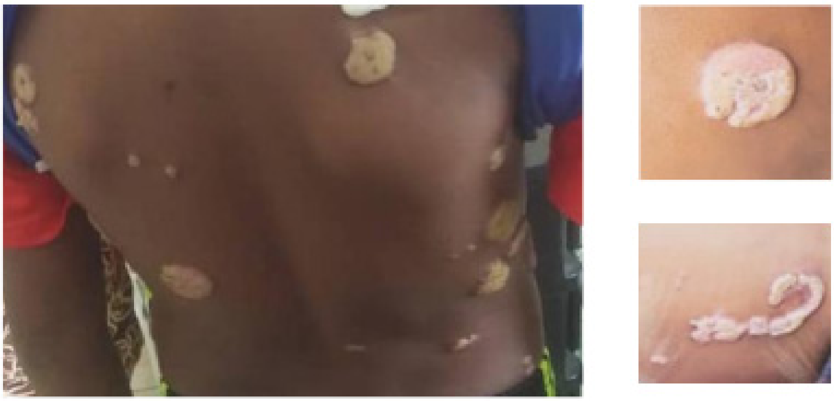

Gambar B: Sporotrichosis

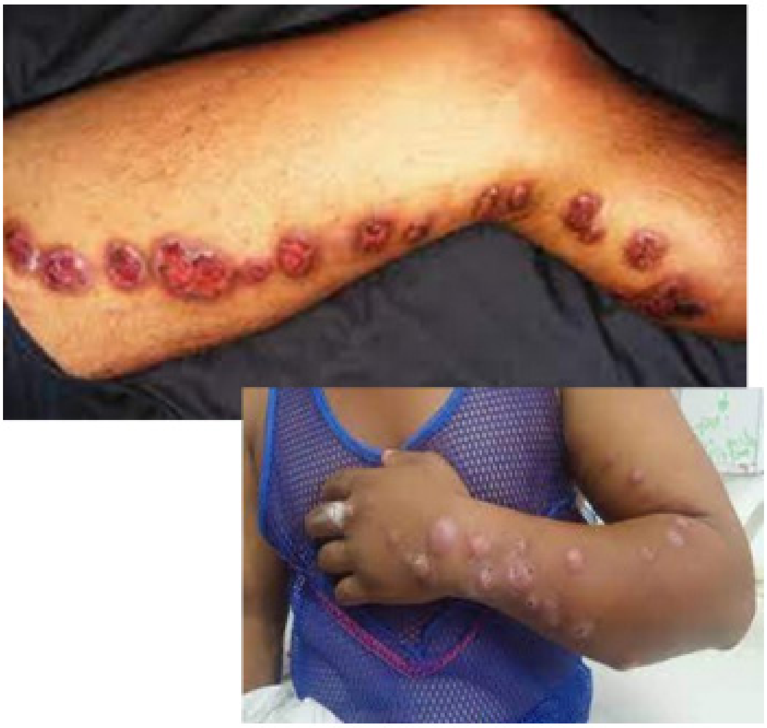

Gambar C: Atlas Kusta

Paucibacillary (PB) Leprosy: 1 to 5 skin lesions.

1.1

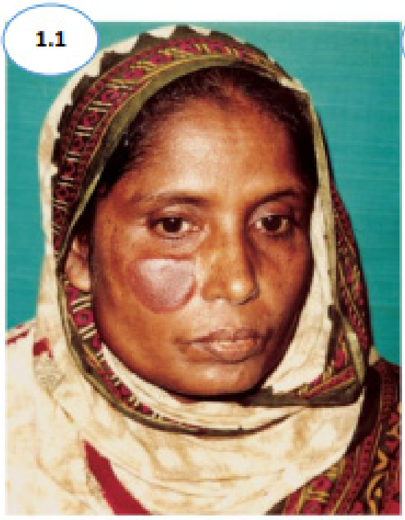

1.2

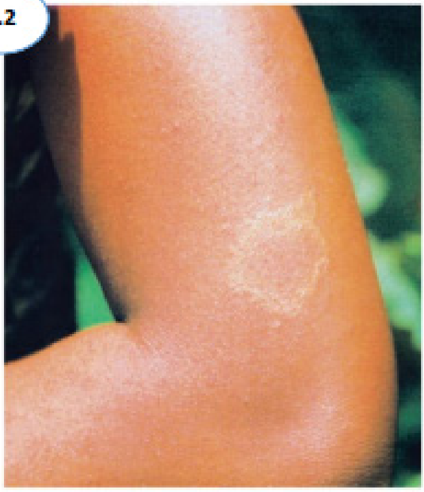

1.3

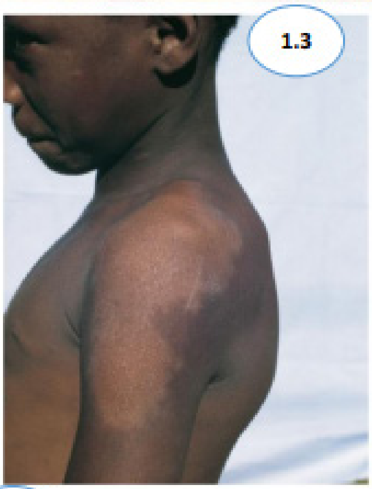

1.1 A single, raised, well-defined lesion on the right cheek with loss of sensation.

1.2 The ring-like lesion. Surface slightly rough and dry, the edge was raised. Loss of sensation to light touch (cotton wool).

1.3 A lesion over the left shoulder with hypopigmentation (reduced colouring) with small 'satellite' lesions near the edge.

1.4 Well-defined lesion on the right buttock and two more lesions on the left.

1.5 Well-defined lesion on the left buttock. The edge raised on palpation. Definite loss of sensation, especially towards the edges, to light touch (cotton wool).

1.4

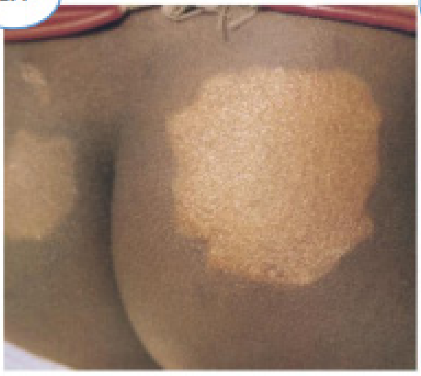

1.5

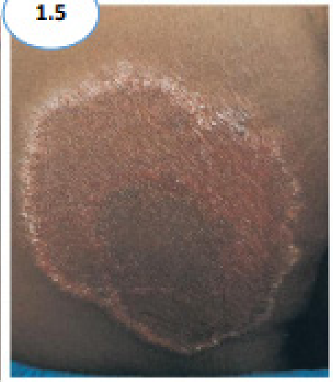

Gambar C: Atlas Kusta

Multibacillary (MB) leprosy: 6 or more skin lesions

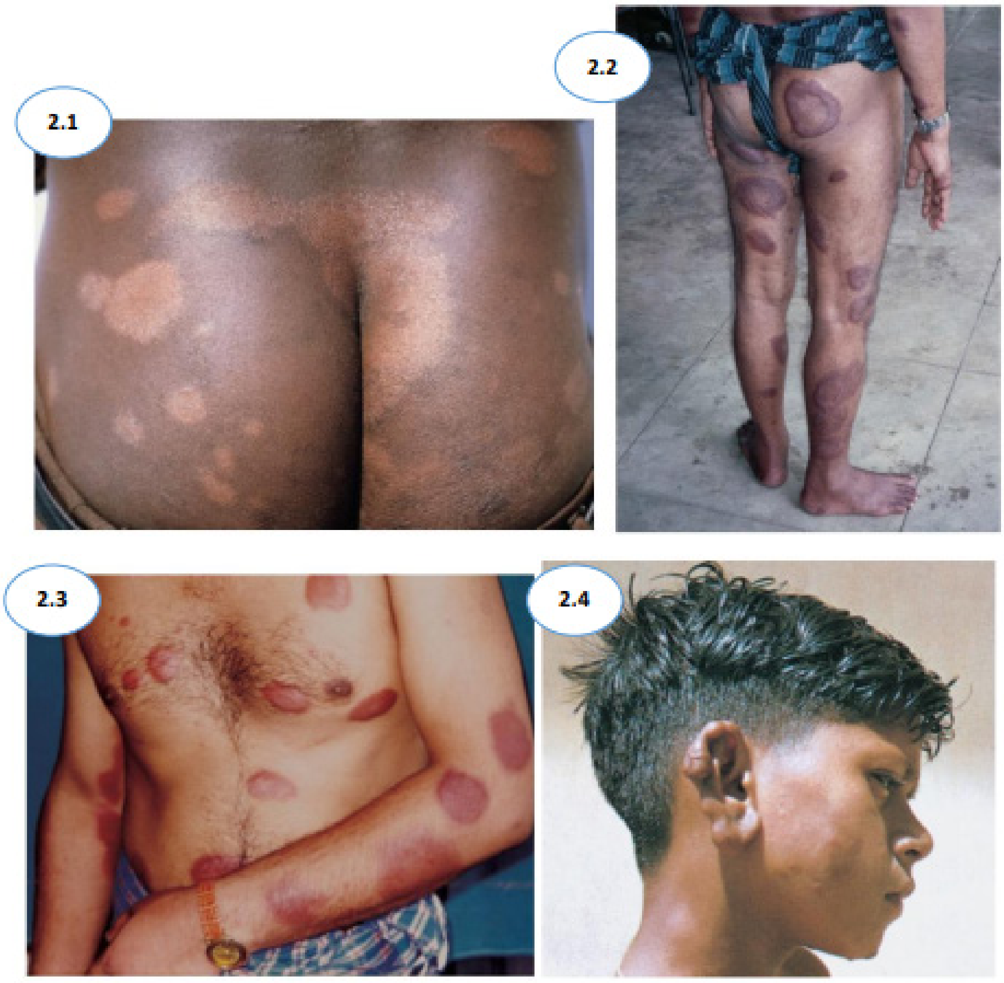

2.1 Numerous hypopigmented lesions over the buttocks and lower back. Some larger patches showed loss of sensation to light touch (cotton wool).

2.2 'Punched out' lesions on the buttocks and legs and similar lesions on the trunk and arms. Most of the lesions loss of sensation to light touch (cotton wool) and there were enlarged peripheral nerves.

2.3 Raised red patches over the trunk and limbs and several on the face. Some peripheral nerves were enlarged. Some patches showed loss of sensation to light touch (cotton wool).

2.4 Patches on the face and neck, and many nodules (small lumps) on the right ear. Always examine the ears for leprosy.

Gambar C: Atlas Kusta (Disability – Deformity)

3.1

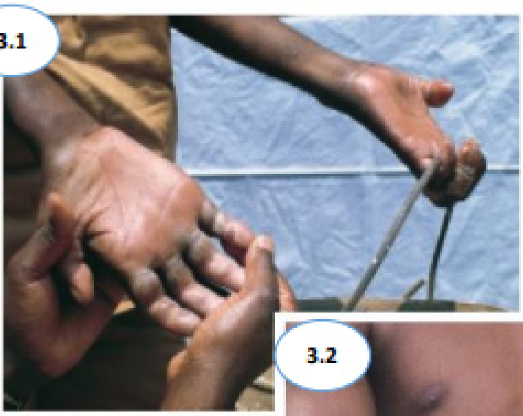

3.1 Bilateral 'claw' hands due to nerve damage, muscle weakness and contractures AND burn and scars of the fingers due to loss of sensation.

3.2

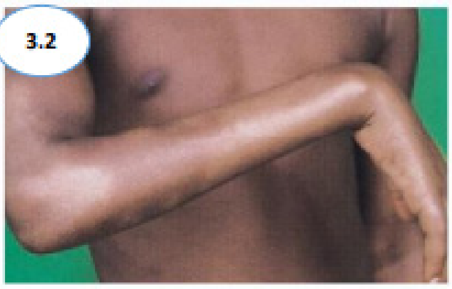

3.2 Wrist drop due to damage to the radical nerve in the upper arm.

3.3

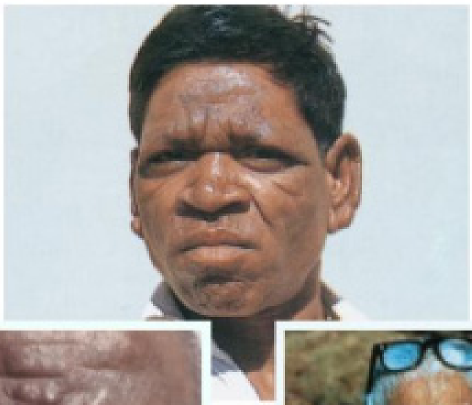

3.3 Thickened (infiltrated) and shiny skin on the face. The ears show infiltration and nodule formation.

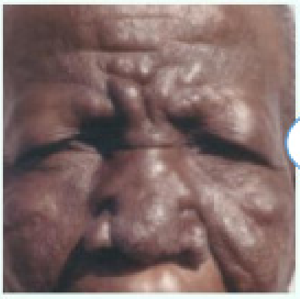

3.4

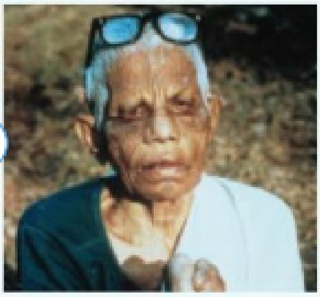

3.4 Upper left; collapse of the cartilage of the nose.

Upper right; Elderly patient with eye complications.

Gambar D: Cacing

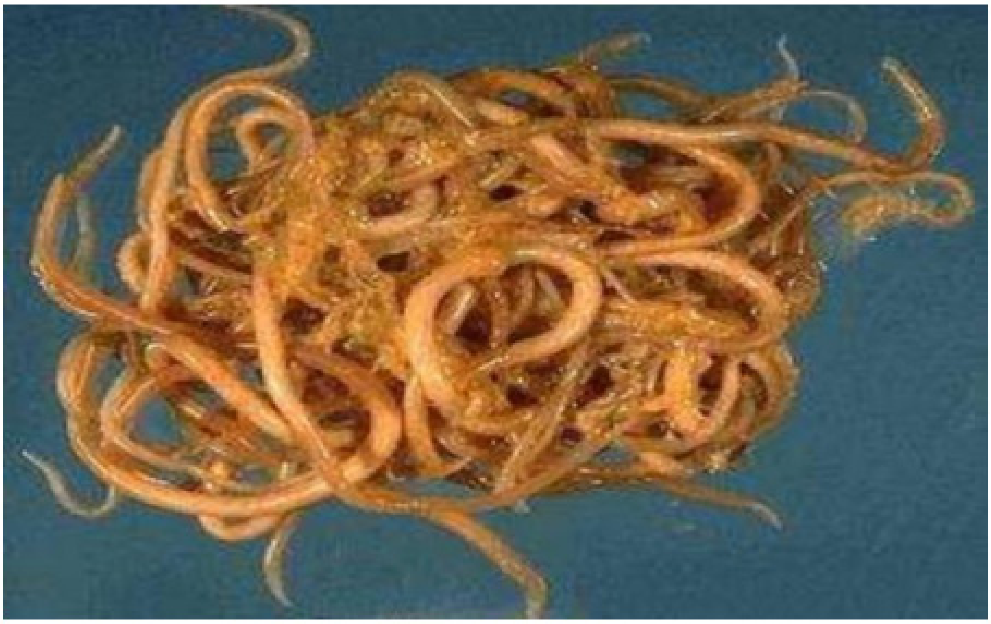

Gambar E: Filariasis

Soalan

Dalam tempoh 12 bulan yang lepas, pernahkah anda melihat individu dengan kaki untut/kaki gajah di kawasan anda tinggal?

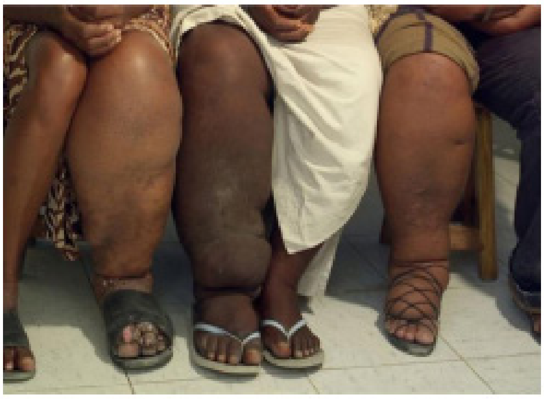

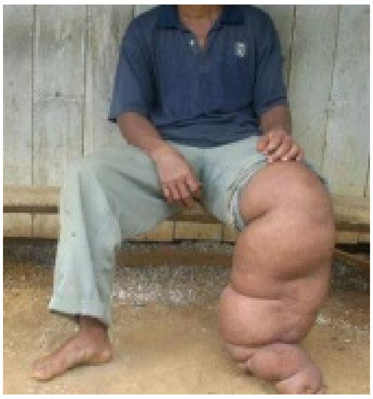

Soalan

Pernahkah anda didapati positif cacing filariasis?

Jawapan:

1. Slaid darah

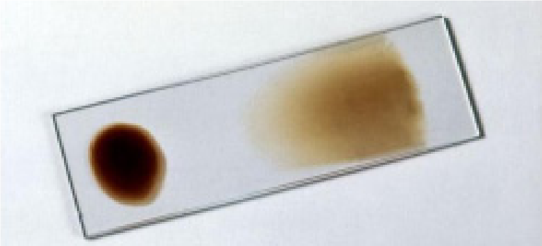

2. Ujian antibodi rapid

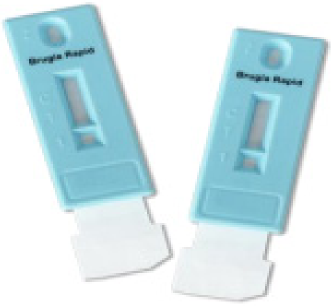

APPENDIX 10: SUMMARY OF PUBLICITY ACTIVITIES AND SAMPLES OF PUBLICITY MATERIALS

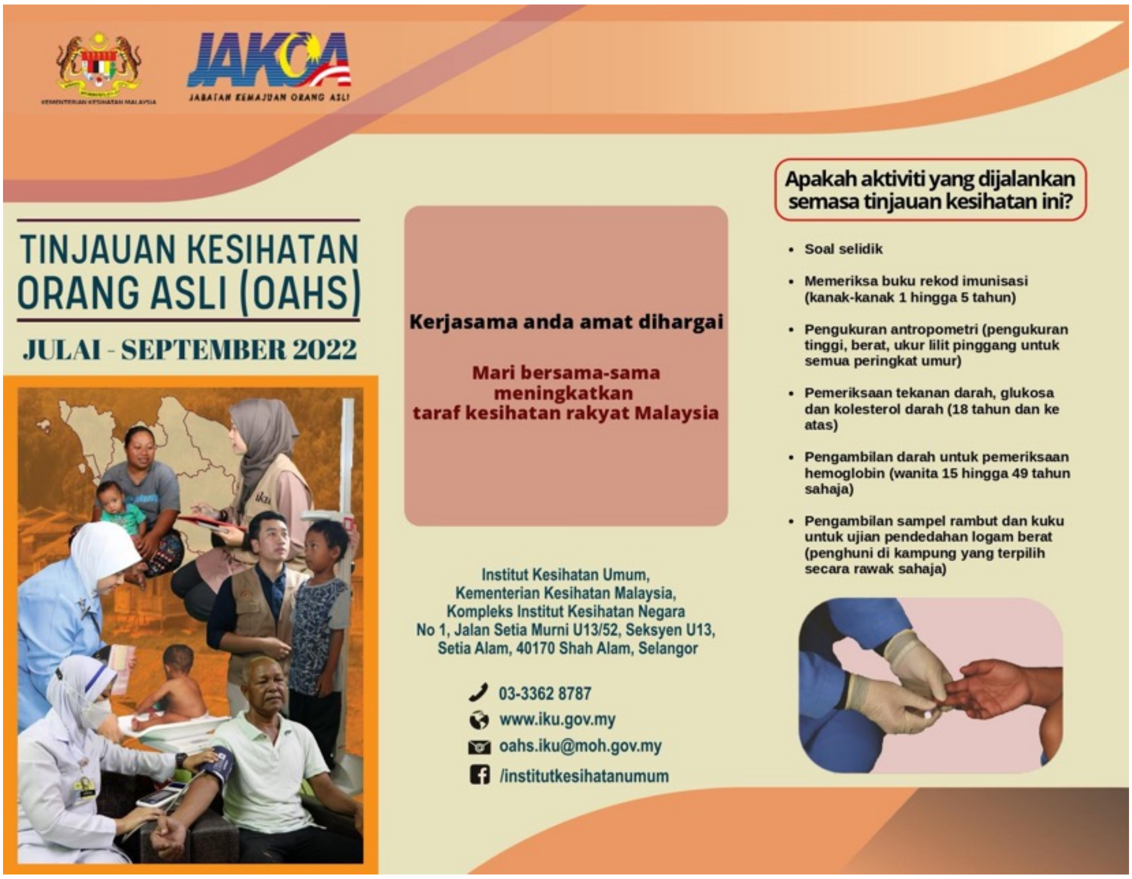

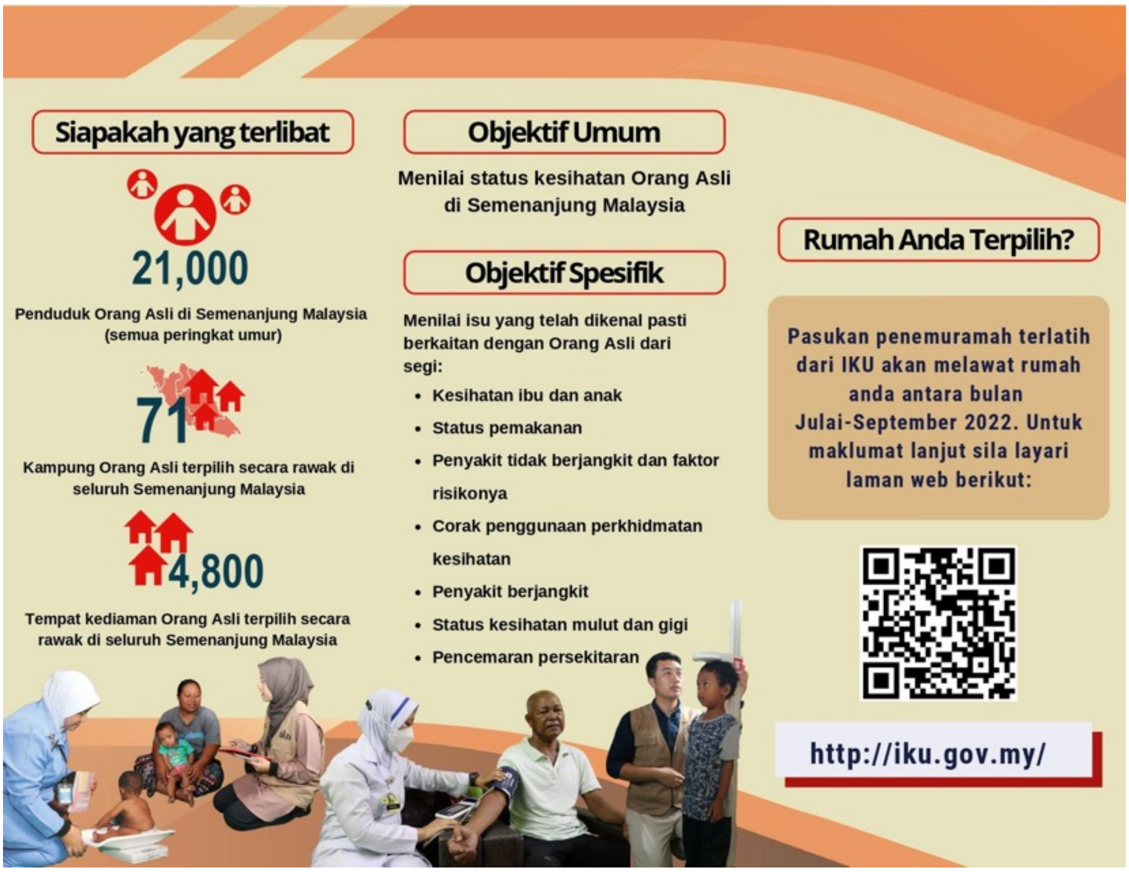

Orang Asli Health Survey Brochure
