# Supplementary material for: Determinants of outpatient service use among Orang Asli in Malaysia using Andersen’s Behavioural Model
Source: PLoS One. 2026 Jan 22;21(1):e0340502. doi: 10.1371/journal.pone.0340502 (PMC12826521; doi:10.1371/journal.pone.0340502)
Supplement: S1 Table — (DOCX) [file pone.0340502.s002.docx]

**S2 Table.** Distribution of outpatient healthcare users in the last 12 months, stratified by tribes and locality, OAHS 2022 (n=1,878)

| Strata | Senoi | | | Proto Malay | | | Negrito | | |
| --- | --- | --- | --- | --- | --- | --- | --- | --- | --- |
|  | Count | Estimated population | % weighted (95%CI) | Count | Estimated population | % weighted ( 95%CI) | Count | Estimated population | % weighted ( 95%CI) |
| Urban | - | - |  | 228 | 336 | 100.0 | - | - |  |
| Fringe | 508 | 8,958 | 61.2 (42.36-77.19) | 156 | 5,215 | 35.6 (20.02-55.02) | 377 | 465 | 3.2 (1.79-5.59) |
| Remote | 316 | 3,367 | 76.5 (58.16-88.40) | 167 | 920 | 20.9 (9.90-38.82) | 126 | 115 | 2.6 (1.38-4.92) |

Note: %: percentage.

Percentage in this table represents the weighted proportion among outpatient users; meanwhile the overall prevalence of outpatient utilisation among Orang Asli adults was 17.9%
