# Supplementary material for: Determinants of outpatient service use among Orang Asli in Malaysia using Andersen’s Behavioural Model
Source: PLoS One. 2026 Jan 22;21(1):e0340502. doi: 10.1371/journal.pone.0340502 (PMC12826521; doi:10.1371/journal.pone.0340502)
Supplement: S2 Table — (DOCX) [file pone.0340502.s003.docx]

**S3 Table.** Distribution of outpatient healthcare users in the last 12 months, stratified by sex and employment, OAHS 2022 (n=1,878)

| Employment status | Male | | | Female | | |
| --- | --- | --- | --- | --- | --- | --- |
|  | Count | Estimated population | % weighted (95%CI) | Count | Estimated population | % weighted ( 95%CI) |
| Unemployed | 131 | 1,279 | 12.26 (9.47-15.73) | 888 | 9,155 | 87.74 (84.27-90.53) |
| Employed | 490 | 4,524 | 50.95 (44.65-57.22) | 366 | 4,356 | 49.05 (42.78-55.35) |
| Missing | 1 | - | - | 2 | - | - |

Note: %: percentage; -: indicates relative standard error (RSE) >30

Percentage in this table represents the weighted proportion among outpatient users, meanwhile overall prevalence of outpatient utilisation among Orang Asli adults was 17.9%.
