# Supplementary material for: Determinants of outpatient service use among Orang Asli in Malaysia using Andersen’s Behavioural Model
Source: PLoS One. 2026 Jan 22;21(1):e0340502. doi: 10.1371/journal.pone.0340502 (PMC12826521; doi:10.1371/journal.pone.0340502)
Supplement: S3 Table — (DOCX) [file pone.0340502.s004.docx]

**S4 Table**. Distribution of outpatient healthcare users in the last 12 months, stratified by tribes and NCD, OAHS 2022 (n=1,878)

| Presence of NCD | Senoi | | | Proto Malay | | | Negrito | | |
| --- | --- | --- | --- | --- | --- | --- | --- | --- | --- |
|  | Count | Estimated population | % weighted (95%CI) | Count | Estimated population | % weighted ( 95%CI) | Count | Estimated population | % weighted ( 95%CI) |
| No NCD | 571 | 8,457 | 62.30 (46.88-75.58) | 406 | 4,642 | 34.19 (21.23-50.05) | 411 | 476 | 3.51 (2.13-5.71) |
| 1 NCD | 125 | 1,869 | 61.13 (41.73-77.54) | 72 | 1,105 | 36.15 (20.10-56.03) | 73 | 83 | 2.73 (1.68-4.40) |
| 2 or more | 128 | 1,999 | 72.86 (55.65-85.17) | 73 | 723 | 26.37 (14.21-43.64) | 19 | - | - |

Note: %: percentage; NCD: non-communicable disease

-: indicates relative standard error (RSE) >30

Percentage in this represents the weighted proportion among outpatient users, while overall prevalence of outpatient utilisation among Orang Asli adults was 17.9%.
